# Comprehensive Insight into the Mechanism, Material Selection and Performance Evaluation of Supercapatteries

**DOI:** 10.1007/s40820-020-0413-7

**Published:** 2020-04-04

**Authors:** Saravanakumar Balasubramaniam, Ankita Mohanty, Suresh Kannan Balasingam, Sang Jae Kim, Ananthakumar Ramadoss

**Affiliations:** 1 School for Advanced Research in Polymers, Laboratory for Advanced Research in Polymeric Materials, Central Institute of Plastics Engineering and Technology, Bhubaneswar, 751024 India; 2 Department of Materials Science and Engineering, Faculty of Natural Sciences, Norwegian University of Science and Technology (NTNU), Trondheim, 7491 Norway; 3 Nanomaterials and Systems Laboratory, Major of Mechatronics Engineering, Faculty of Applied Energy System, Jeju National University, Jeju, 63243 Republic of Korea

**Keywords:** Supercapattery, Energy density, Power density, Redox materials, Carbon materials

## Abstract

Electrochemical energy storage devices (EESs) play a crucial role for the construction of sustainable energy storage system from the point of generation to the end user due to the intermittent nature of renewable sources. Additionally, to meet the demand for next-generation electronic applications, optimizing the energy and power densities of EESs with long cycle life is the crucial factor. Great efforts have been devoted towards the search for new materials, to augment the overall performance of the EESs. Although there are a lot of ongoing researches in this field, the performance does not meet up to the level of commercialization. A further understanding of the charge storage mechanism and development of new electrode materials are highly required. The present review explains the overview of recent progress in supercapattery devices with reference to their various aspects. The different charge storage mechanisms and the multiple factors involved in the performance of the supercapattery are described in detail. Moreover, recent advancements in this supercapattery research and its electrochemical performances are reviewed. Finally, the challenges and possible future developments in this field are summarized.

## Highlights

- This article reviewed the recent progress on material challenges, charge storage mechanism, and electrochemical performance evaluation of supercapatteries.
- Supercapatteries bridge the gap between supercapacitors (low energy density) and batteries (low power density).
- The importance of the design and configuration of the supercapatteries are briefly reviewed and the future direction in this field also outlined at the end.

## Graphical abstract

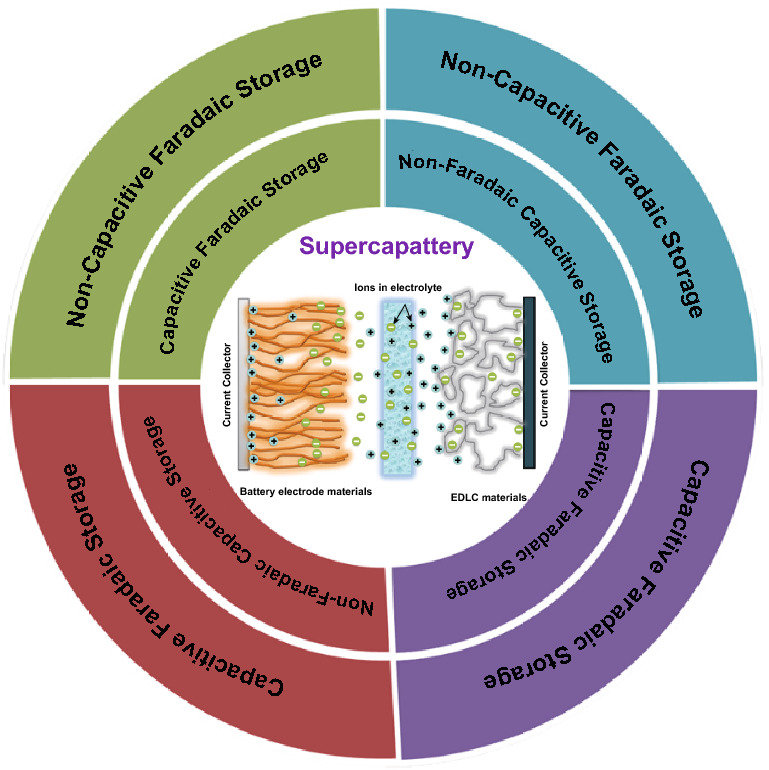

## Introduction

Energy is a mandatory entity for the survival of whole universe. The different forms of energy are used for different purposes. Particularly, the electrical energy is the heart of all human-made things, which is essential for the sustainability of mankind. A lot of advancements in the technological development and miniaturized devices have made the human life as simple, easier and more comfortable. The electrical energy plays an important role in the modern lifestyle of humanity, and mostly, it is obtained from either renewable (wind [1], thermal [2], solar [3], nuclear [4]) or non-renewable sources (coal [5], oils [6], etc.) using different conversion technologies. The demand of electrical energy is increasing day-by-day due to the inflating number of electronic devices and human beings [7–9]. In addition to that, researchers are looking for new types of renewable energy conversion devices to reduce the pollution and environmental disorders, which can make our living place sustainable [10]. Electrical energy conversion from renewable sources is spasmodic; hence, intermediate energy storage devices are essential for the uninterrupted and continuous supply of energy. The electrochemical energy storage (EES) devices play a significant role in electrical and electronic devices with high performance and affordable price [11, 12]. Heterogeneity in the form and application of energy demands the development of energy storage technologies in multiple dimensions. To meet out the demand for high energy and power density of electrochemical energy storage devices, the material development plays a dramatic role [13, 14]. Comprehensively, various EES devices are available; however, batteries [15–18] and supercapacitors [19–21] are considered as two main classes of EES devices due to their high energy and power densities [12, 22–26]. In the view of safety and life cycle, supercapacitors headed over the batteries [27, 28], but they are backward in the energy density [29]. The Li-ion batteries (LIBs) have higher energy density range of ~ 150–200 Wh kg^−1^ [30, 31], which is even higher than that of other types of the batteries such as Ni–Cd [32, 33], Ni–MH [33], lead–acid [34] and so on. Nevertheless, the supercapacitors have higher power density (10 kW kg^−1^) with long life cycle and fast charge/discharge capability [26]. However, the low power density (< 1 kW kg^−1^) of batteries [31, 35] and the very low energy density of supercapacitors (5–10 Wh kg^−1^) [36] hinder their practical applications in hybrid electric vehicles, renewable energy storage grid and so on. Therefore, more attention should be given to the development of safe, long cycle life and high-performance energy storage devices having both high energy and power densities [12, 37–42].

**Fig. 1:**
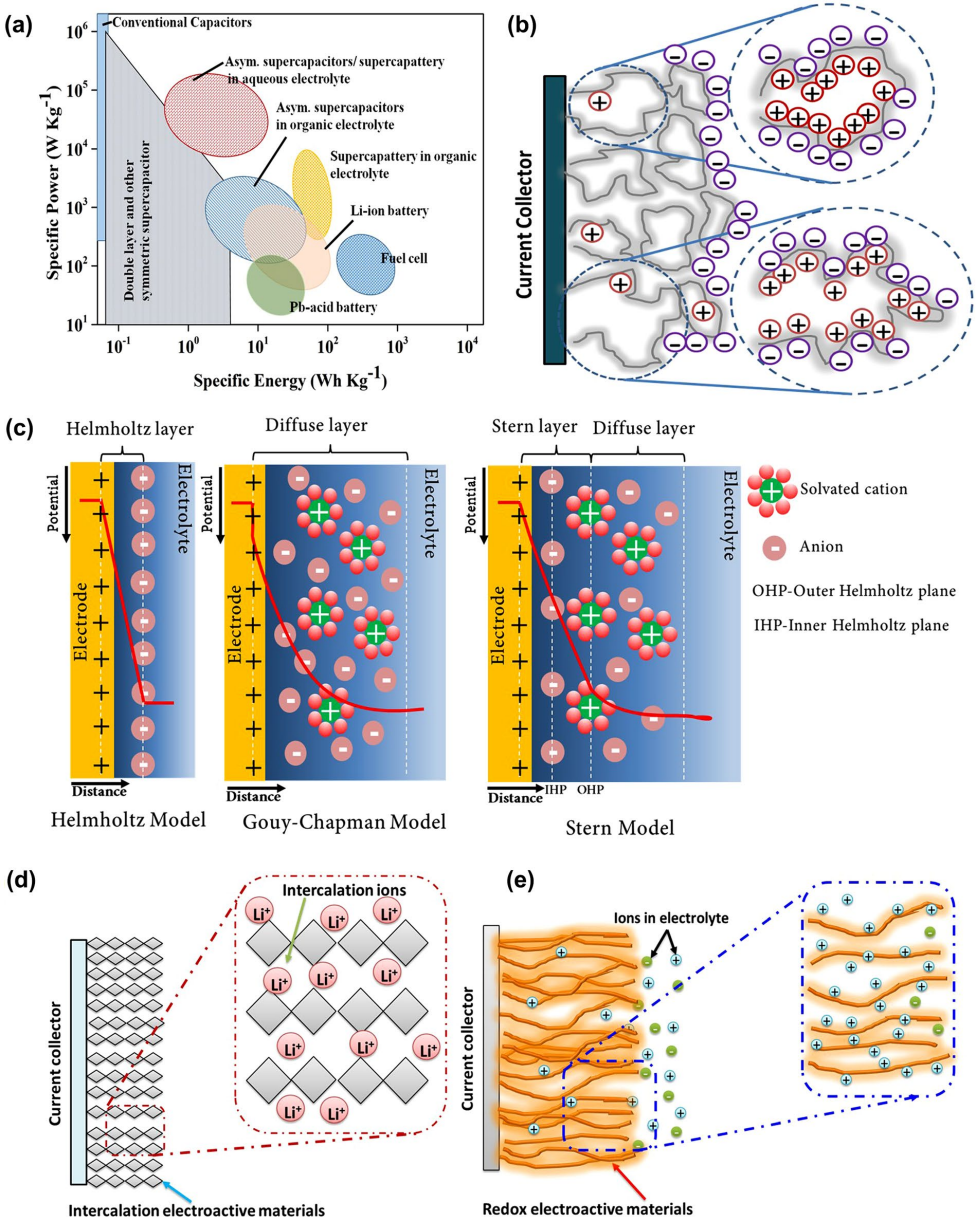
**a** Ragone plot of various electrochemical energy conversion and storage devices [43]. **b** Schematic illustration of charge storage mechanism of EDL capacitor in porous carbon electrode. **c** Representation of EDLC structures: Helmholtz model, Gouy–Chapman model and Gouy–Chapman–Stern model. Schematic representation of the charge storage mechanisms in pseudocapacitor; **d** Intercalation (bulk redox) and **e** surface redox

Figure 1a represents the clear picture of energy and power densities of various energy storage devices known as Ragone plot [43–48]. It is clearly indicating that the conventional and electric double-layer capacitor (EDLC)-based supercapacitors have higher power density than all types of batteries but inferior in energy density. Moreover, LIBs have higher energy density when compared to all other batteries as well as supercapacitors [49, 50], but they have a low power density [43, 51]. Interestingly, different kinds of asymmetric supercapacitors/supercapatteries [51] showed moderate range of energy and power densities, which is almost similar or superior to that of the existing LIBs. Therefore, asymmetric supercapacitors/supercapatteries have gained more interest in the energy storage applications due to their comparable performance with LIBs as well as safer [52] and eco-friendly nature [53]. For the further development of energy storage devices, it is significantly important to understand the working mechanism as well as in-depth knowledge in the configuration of supercapatteries. This review describes briefly about the evolution of supercapattery from the supercapacitor and battery. Further, it describes about the various energy storage mechanisms adapted in the supercapattery research with the aid of electrochemical studies. Moreover, various parameters in the construction of supercapatteries such as material selection, electrode fabrication, device configuration and its electrochemical analysis have been discussed in detail. The detailed review of the literature related to the design and fabrication of supercapatteries is briefed. Finally, the existing challenges in supercapattery design, development and the future research prospective are highlighted in this review.

## Charge Storage Mechanism of Supercapacitors

Supercapacitors are classified into two types [44–48] based on their energy storage mechanisms: electric double layer capacitor (EDLC) [54, 55] and pseudocapacitor [56, 57].

### Electric Double-Layer Capacitor

The EDLC shows an outstanding power density due to very fast adsorption and desorption of electrolyte ions at the electrode/electrolyte interface which forms the electric double layer while charging/discharging of the device (Fig. 1b) [58]. In 1853, Hermann von Helmholtz proposed the first model for EDL capacitance (Fig. 1c). According to this model, the electrical conductor placed in an electrolyte in the presence of electric field forms the electric double layer at electrode/electrolyte interface through electrostatic force, which is known as Helmholtz layer. Moreover, no charge transfer occurs on the formation of this layer and the separation of charge is mainly due to the electrostatic force. Further, Gouy and Chapman extended this model by introducing a diffusion layer (Fig. 1c), which arises due to the thermal motion of ions in the electrolyte. However, this model was failed when highly charged double layers form at the electrode/electrolyte interface due to either more highly charged electrode or the high concentration of electrolyte ions. Later, Stern proposed a new model by merging Helmholtz, Gouy and Chapman models and it is represented in Fig. 1c [59]. Based on this model, the Stern and diffuse layers are considered for the capacitance in EDLC. Also, Stern/compact layer was divided into two planes, such as inner Helmholtz plane (IHP) at closer to the electrode as it passes through the center of the specifically adsorbed ions on the electrode surface, whereas outer Helmholtz plane (OHP) is at the distance of closest approach from the electrode surface as it passes through the center of solvated ions. In 1957, Becker developed the first EDLC for practical applications using carbon-based materials. Mostly, the carbon and its allotropic form of materials have been used for EDL capacitor applications owing to their high surface area, meso-/microporous structure, low cost, eco-friendliness, higher electrical conductivity, chemical and thermal stability. The detailed EDLC storage mechanism in porous carbon electrode is described in Fig. 1c. Firstly, when a thin atomic inner Helmholtz layer forms on the surface of porous carbon, it gets in contact with electrolyte, through non-solvated or very weak solvated ions, so it becomes highly permeable to ions movement. Secondly, a thicker outer Helmholtz layer is formed by the solvated ions through strong electrostatic interaction. Further, this layer gets extended to a broad layer by solvated ions through a thermal motion and is known as Gouy–Chapman diffuse layer. The formation of broad layer results in small potential difference between the Gouy–Chapman diffuse layer and Helmholtz layers described as zeta potential (ξ-potential), which indicates the degree of charge storage [60].

### Pseudocapacitors

The pseudocapacitor is another type of supercapacitor, which stores the energy through the reversible Faradaic reaction or surface-based redox reaction, which occurs at the electrode surface. The electrochemical characteristics of these devices resemble the capacitive signature [61]. It has entirely different energy storage mechanism than EDLC. Generally, it stores the charge by both the reversible redox reaction and the electrochemical adsorption/desorption [62], i.e., intercalation/de-intercalation and doping/de-doping of ions at the electrode/electrolyte interface through Faradaic charge transfer process [39]. The pseudocapacitor delivers higher capacitance and energy density than EDLC due to surface active redox reaction and discharges (within few seconds) the energy much faster than batteries. In surface redox pseudocapacitance, the ions are adsorbed on or near surface of the materials due to Faradaic charge transfer.

However, in intercalation, the ions are tunneled through layers or intercalate within the electrode materials during Faradaic transfer without changing its inherent crystal structure or phase [63]. Battery-type materials usually obey this intercalation-based redox reaction [63]. Further, the charge storage mechanism of pseudocapacitor is schematically described in Fig. 1d, e. It has superior benefits in the aspects of energy storage via chemical reactions within the bulk material [62, 64, 65]. Figure 1 represents the detailed description of various charge storage mechanisms in supercapacitor. Typically, most of the transition metal oxides and conducting polymers are coming under the category of pseudocapacitive materials. The charge storage can be generalized such as capacitive non-Faradaic (EDLC), capacitive Faradaic (pseudocapacitive) and non-capacitive Faradaic (battery type).

## Supercapattery

Another class of supercapacitor is supercapatteries, which consists of high-power EDLC electrode at one side and high-energy density battery-type electrode at other side. It is also called as hybrid supercapacitor or asymmetric supercapacitor or battery–supercapacitor hybrid device, etc. The asymmetric supercapacitor describes about the assembly of two different kinds of electrode materials in positive and negative electrode sides in aqueous or non-aqueous electrolytes. These terms are commonly used before the introduction of some specified terminologies such as supercapattery, hybrid-ion capacitor or battery–supercapacitor hybrid device. The alkaline-ion hybrid supercapacitors or battery–supercapacitor hybrid device are also called as a hybrid supercapacitor, in which the alkaline ions are mostly used for intercalation/de-intercalation process. Further, it can be classified based on the types of alkaline electrolytes used for energy storage such as lithium ion, sodium ion and potassium ion. In asymmetric cell, the pseudocapacitive materials and battery-type materials are usually used as a positive electrode and mostly carbon-based materials (EDLC) or a few negative potential metal oxides (Fe_2_O_3_, Bi_2_O_3_, MoO_3_) are used as a negative electrode. In hybrid asymmetric cell (or) supercapattery or supercabattery devices, mostly the battery-type electrodes are used as positive electrode to construct these devices. Supercapattery (= supercapacitor + battery) has been announced as a new term to signify a vast range of devices that exploit both capacitive and non-capacitive Faradaic charge storage mechanisms at either level of electrode materials [39, 66]. The supercapattery merges the gap between battery and supercapacitor with improved energy and power densities [67]. Basically, this device is hybridization of both capacitive and Faradaic charge storage mechanisms in a single device in order to achieve the optimized energy density as well as power density. For the assembly of a supercapattery device, there are four possibilities:

(i) Capacitive Faradaic system + Capacitive non-Faradaic system (pseudocapacitive + EDLC).
(ii) Capacitive Faradaic system + Capacitive Faradaic system (pseudocapacitive + pseudocapacitive).
(iii) Capacitive non-Faradaic system + Non-capacitive Faradaic system (EDLC + battery).
(iv) Capacitive Faradaic system + Non-capacitive Faradaic system (pseudocapacitive + battery).

The details of different possible assemblies of electrodes (according to their charge storage mechanisms) for supercapacitors such as symmetric and asymmetric integration are given in Table 1 [43].

**Table 1:** Classification of various energy storage devices according to their charge storage mechanisms

Device		Supercapattery				Battery
	Supercapacitor			Hybrid supercapacitor		
	EDLC	Pseudocapacitors				
Electrode material	NFCS + NFCS	NFCS + CFS	CFS + CFS	NFCS + NCFS	CFS + NCFS	NCFS + NCFS

*NFCS* non-Faradaic capacitive storage = EDLC storage, *CFS* capacitive Faradaic storage = pseudocapacitive storage, *NCFS* non-capacitive Faradaic storage = battery-type storage

**Fig. 2:**
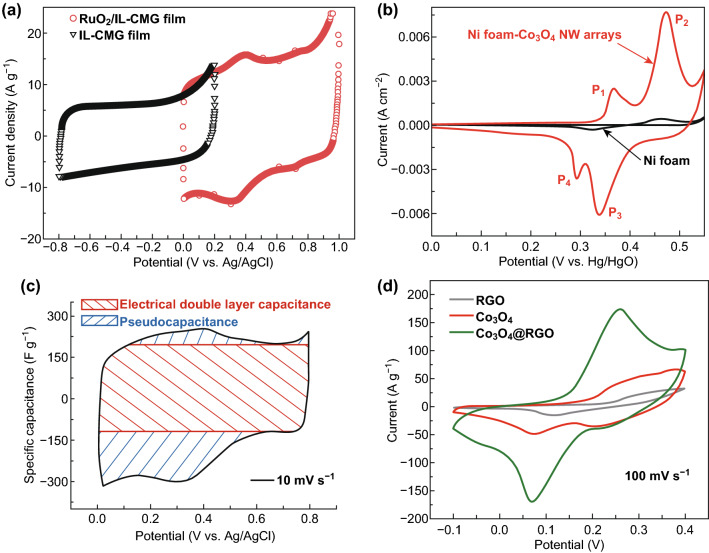
Typical cyclic voltammetry profiles of **a** capacitor electrode [EDLC (IL-CMG film) and pseudocapacitance (RuO_2_/IL-CMG film)]. Adapted with permission from Ref. [68]. Copyright 2012 The Royal Society of Chemistry. **b** Battery-type electrode (Ni foam–Co_3_O_4_ NW arrays). Reprinted with permission from Ref. [69]. Copyright 2011 Elsevier. **c** Composite electrode (EDLC + pseudocapacitive composite electrode). Reproduced with permission from Ref. [70]. Copyright 2011 American Chemical Society and **d** composite electrode (supercapattery [Co_3_O_4_@rGO]: EDLC [rGO] + battery [Co_3_O_4_] composite electrode) in a three-electrode configuration Adapted with permission from Ref. [71]. Copyright 2016 Elsevier

The energy storage mechanisms of different electrode materials are clearly distinguishable by electrochemical measurements such as cyclic voltammogram (CV) and galvanostatic charge–discharge (GCD) (figure is not shown here). The EDLC and pseudocapacitive materials display almost a rectangular shape for ionic liquid functionalized chemically modified graphene (IL-CMG) film (black color CV plot in Fig. 2a) and quasi-rectangular for RuO_2_/IL-CMG film (red color CV plot in Fig. 2a) shape in CV measurement and in GCD, linear or a slightly deviated nonlinear time-dependent change of voltage at a constant current. The slight deviation in the GCD curve is due to the combination of both double-layer capacitance and pseudocapacitance. For example, graphene is a well-known EDLC material which displays a rectangular shape in the CV curve and linear time-dependent changes of voltage in GCD curves, respectively [68]. All the carbon families are coming under the belt of EDLC. MnO_2_, RuO_2_ and PANI are good examples of pseudocapacitive materials for energy storage applications. Moreover, CV, GCD curves of pseudocapacitive materials look like EDLC materials (black color CV of IL-CMG film in Fig. 2a), but the dominant energy storage is contributed by the reversible surface redox reaction (broad humps as shown in red color CV of RuO_2_/IL-CMG film) at the surface of the electrode through insertion/de-insertion or doping/de-doping process in neutral or acidic electrolyte without change of its crystal phase [39].

In contrast, battery-type materials display an entirely different CV and GCD profile, in which a higher current density near the inherent oxidation–reduction (redox) potentials (clear peaks in CV) suggests that the charge is stored in this material through reversible Faradaic redox reaction (non-capacitive) through crystal phase transition. Similarly, the voltage plateau appears in the GCD curve due to the existence of different phases. Mostly, these types of electrode materials go through the bulk redox reaction.

The Co_3_O_4_-based materials are the best example for battery-type electrodes. The CV curve of this material (red color CV plot of Co_3_O_4_ in Fig. 2d and red color CV plot of Ni foam–Co_3_O_4_ NW arrays in Fig. 2b) clearly shows the distinct oxidation and reduction peaks in alkaline electrolyte confirming the reversible Faradaic reactions [69]. Similarly, the composites of EDLC material with battery-type material and pseudocapacitive materials show both EDLC as well as pseudocapacitive/battery-type behaviors in the CV as well as GCD curves. The functionalized graphene is the best example for composite electrode with EDLC and pseudocapacitive behavior and the CV (black color CV plot of rGO in Fig. 2d) curves are looking like EDLC/pseudocapacitors [70]. Similarly, the composite made by EDLC and battery-type materials such as Co_3_O_4_/rGO composite electrode [71] displays both the behaviors like EDLC as well as Faradaic process, but Faradaic process is more dominant than EDLC (green color CV plot of Co_3_O_4_@rGO in Fig. 2d). Moreover, EDLC materials acted as a conducting path for the electrons in the composite electrode. The mixed nature of the capacitor and the battery is represented in the CV of Co_3_O_4_@ rGO, where rGO shows EDLC property and Co_3_O_4_ displays battery-type property in Fig. 2d. The CV curves demonstrate the features of both batteries and supercapacitors behavior. Further, it can be named as either supercapattery/asymmetric supercapacitor (with behavior close to that of a supercapacitor, Fig. 2c) or supercabattery (displaying behavior close to that of a battery, Fig. 2d).

**Fig. 3:**
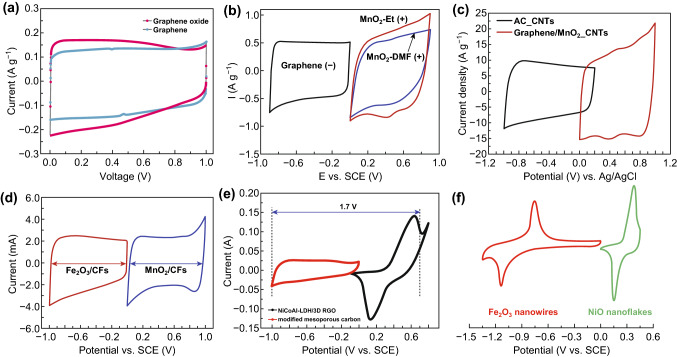
Typical cyclic voltammetry curves of different device configurations: **a** symmetric supercapacitor (EDLC {Graphene} and pseudocapacitive {graphene oxide}). Reprinted with permission from Ref. [72]. Copyright 2011 The Royal Society of Chemistry. **b** Asymmetric supercapacitor (EDLC{graphene}║pseudocapacitive {MnO_2_-DMF and MnO_2_-Et}). Reproduced with permission from Ref. [73]. Copyright 2015 Elsevier. **c** Asymmetric supercapacitor (EDLC {AC_CNTs}║EDLC + pseudocapacitive {graphene/MnO_2__CNTs}). Reproduced with permission from Ref. [74]. Copyright 2012 The Royal Society of Chemistry. **d** Asymmetric supercapacitor (EDLC + pseudocapacitive {Fe_2_O_3_/CFs}║EDLC + pseudocapacitive{MnO_2_/CFs}). Adapted with permission from Ref. [75]. Copyright 2018 Elsevier. **e** Hybrid supercapacitor/supercapattery (EDLC║battery-type). Reprinted with permission from Ref. [76]. Copyright 2017 Elsevier and **f** supercapattery (pseudocapacitive {Fe_2_O_3_ nanowires}║battery-type {NiO nanoflakes}). Reproduced with permission from Ref. [77]. Copyright 2015 The Royal Society of Chemistry

As it is observed before, the electrochemical measurement like CV, GCD are clearly distinguishable based on the charge storage mechanisms. However, it is important to study these materials, in full cell (two-electrode system), to show their capability for real-time applications. A full cell consists of two electrodes namely positive electrode (cathode) and a negative electrode (anode). Based on the assembly of these electrodes, it is further classified into symmetric and asymmetric cells. The symmetric cell is assembled with same material for both positive and negative electrodes, and mostly EDLC, pseudocapacitive materials are used. For example, symmetric supercapacitors are constructed by using graphene (EDLC type) as an electrode material for both the positive and negative electrodes and graphene oxide (pseudocapacitive type) is used as both positive and negative electrodes of a symmetric supercapacitor. The full cell of graphene electrodes showed a rectangular behavior in CV (blue color CV plot in Fig. 3a), and graphene oxide showed a quasi-rectangular shape (red color CV plot for graphene oxide in Fig. 3a), which are more similar behavior observed in the individual electrodes in three-electrode configuration [72]. In asymmetric cell, the pseudocapacitive materials and battery-type materials are usually used as a positive electrode and mostly carbon-based materials (EDLC) or a few negative potential metal oxides (Fe_2_O_3_, Bi_2_O_3_, MoO_3_) are used as a negative electrode. In hybrid asymmetric cell (or) supercapattery or supercabattery devices, mostly the battery-type electrodes are used as positive electrode to construct these devices.

The MnO_2_║Graphene is an asymmetric cell with pseudocapacitive positive and EDLC negative electrodes [73]. The asymmetric device configuration showed a quasi-rectangular shape CV (Fig. 3b) at positive, which clearly indicates the pseudocapacitive behavior and rectangular shape at negative electrode indicates the EDLC behavior. Similar behavior was observed in the EDLC-pseudocapacitive composite material-based cell (Fig. 3c, d) [74, 75]. In supercapattery, a clear redox peak is observed in the CV and a nonlinear GCD curves with voltage plateau, which indicates the reversible Faradaic reaction during the electrochemical reaction. The NiCoAl-LDH/3D rGO║mesoporous carbon [76] and NiO║Fe_2_O_3_ [77] are the two examples of supercapattery, and its corresponding CV curves of both supercapattery cells are shown in Fig. 3e, f, respectively.

## Factors Affecting the Performance of Supercapattery

The ultimate aim of the supercapattery is to match the performance between the supercapacitor and battery with optimized energy and power densities [65]. The energy and power densities are key parameters to evaluate the performance of energy storage devices. The energy density value mainly depends on capacitance and potential window as well as the internal resistance of the device, which is represented in Eq. 1 [78].

1 $$E = \frac{1}{2}CV^{2}$$

where, *E* is the energy density and *C* and *V* are the capacitance and potential window of the device, respectively.

Therefore, these three parameters play vital roles in the designing of high-performance cell. Along with the optimized energy and power densities, a sustainable supercapattery device demands for appreciable cyclic stability or capacitance retention over many charge/discharge cycles with higher rate capability.

The charge balance between the positive electrode (positrode) and a negative electrode (negatrode) is necessary to accommodate the electrochemical reaction at both ends of the devices. Therefore, it is important to balance the charge between the positive electrode and the negative electrode during the assembling of device. The expression of charge stored at the electrode material in the supercapattery is given by Eq. 2 [79]:

2 $$Q = C \times \Delta V \times m$$

where, *C* is the specific capacitance, $\Delta V$ is the working potential window, and m is the mass of the electroactive material.

According to the law of conservation of charge (Eq. 3) [199],

3 $$Q^{ + } = Q^{ - } = > C^{ + } \times\Delta V^{ + }\times m^{ + } = C^{ - }\times \Delta V^{ - }\times m^{ - }$$

The mass ratio of the electrode is calculated from Eq. 4:

4 $$\frac{{m^{ + } }}{{m^{ - } }} = \frac{{C^{ - } \Delta V^{ - } }}{{C^{ + } \Delta V^{ + } }}$$

where *Q*^+^ and *Q*^−^ are the total charge stored at positive and negative electrodes, respectively, $C^{ + }$ and $C^{ - }$ are the specific capacitances of the positive and negative electrodes, respectively, and $\Delta V^{ + } \;{\text{and}}\;\Delta V^{ - }$ are the potential windows of positive and negative electrode materials, respectively.

Here, the specific capacitance of the device/electrode is calculated from galvanostatic charge/discharge curves using Eq. 5 [79]:

5 $$C_{T} = \frac{I}{{ \left( { - \frac{{{\text{d}}V}}{{{\text{d}}t}}} \right)}}\left( {\frac{1}{{m^{ + } }} + \frac{1}{{m^{ - } }}} \right)$$

here, *I* signifies the discharge current and $\frac{{{\text{d}}V}}{{{\text{d}}t}}$ represents the slope of discharge curve in GCD after IR drop.

Consequently, the materials with higher surface area, conductivity and porosity need to be chosen to achieve a higher capacitance. Similarly, the operating potential of the cell highly depends on the electrode materials and types of electrolyte [80].

Further, the potential window (*E*) is calculated from Eq. 6 [81],

6 $$E = E_{0} + \Delta E_{1} + \Delta E_{2} = 1/F(\omega^{\beta } - \omega^{\alpha } )N_{\text{A}} + \Delta E_{1} + \Delta E_{2}$$

where, $\Delta E_{1}$ and $\Delta E_{2}$ are surface dipoles, $\omega^{\alpha }$ and $\omega^{\beta }$ are work function of positive and negative electrode materials, respectively, and $N_{\text{A}}$ is the Avogadro number.

From Eq. 6, it is clearly visible that the work function of both electrodes plays an essential role in the extension of working potential of full cell. The selection of higher work functional electrode materials is another way to increase the cell capacitance through extending the potential window. Mostly, the metal oxides have higher work function, but the oxygen defects in the crystal structure considerably reduce its work function by n-doping, which shifts the Fermi level closer to the conduction band and it is compensated by heat treatment in an oxygen environment. The chemisorption of proton and hydroxide ions on the surface of metal oxides further extends the potential window by modifying the work function of electrodes.

Apart from the work function of electrode materials, electrolyte is also another factor to decide the cell voltage (V). Generally, there are various types of electrolytes used in supercapacitor application such as aqueous, non-aqueous (ionic liquid and organic)-type electrolytes, which decide the cell operating potential. The aqueous electrolyte-based supercapatteries possess good conductivity, cost effectiveness along with environmental benignity [12]. However, the limit of the most aqueous electrolytes is a low decomposition voltage of water, i.e., 1.23 V [12]. Hence, the most of aqueous electrolytes-based supercapatteries operate in the potential window of 0–1.2 V. In order to maximize the potential window, the mass balancing between positive and negative electrode must be done in account of their specific capacitance values (Eq. 3) [82, 83]. Therefore, aqueous electrolytes come with some pros and cons. Similarly, non-aqueous organic electrolytes provide wide potential window as well as temperature range (3.5 V, − 50 to 70 °C) [84]. But their demerits are high flammability and cost. Similarly, ionic electrolytes enable supercapattery device to operate in the potential range of ∼ 4–6 V and they possess low flammability and volatility [85, 86]. However, they are highly viscous at room temperature, which directly affects their ionic conductivity [12]. Furthermore, in high temperature condition ionic electrolytes work well.

The power density is another major factor to decide the performance of supercapattery. Merely it depends on voltage and equivalent series resistance ($R_{\text{s}}$) of the cell. The minimization of the equivalent series resistance of the cell is another factor to maximize the power density, and it can be achieved by reducing the contact resistance between the active material and current collector. Hence, it must be achieved by the uniform deposition of active material on the current collector.

The cycle stability of the electrode is considered as an important parameter in the performance of the EES device because it is the life cycle of the device. There are many factors affecting the cycle stability of the electrode, more importantly the structural integrity, strain relaxation and electrical conductivity of the electrodes during the cycles. Battery-type electrode materials possess classic semi-infinite diffusion (i.e., *i∼ν*^0.5^), whereas supercapacitors obey linear relation between current (*i*) and scan rate (*ν*), i.e., i∼ν [87]. Thus, both kinetic and structural properties of materials are linked with their phase transformations. Upon ion insertion during charge–discharge process, the electrode material goes through dimensional variation which leads to strain [30]. Consequently, phase transformation takes place which comes with significant volume change [30], which affects the integrity of electrode materials negatively. Hence, the cycling stability of device becomes feeble in which redox-based materials are used [30]. Composites of redox materials with carbon-based materials are efficient ways to enhance their stability, which can be done through direct carbonization of redox materials [88–90]. Use of nanostructured materials can be an effective way to enhance the ion accessibility of electrolytes through electrode surface, as they possess very good aspect ratio. Thus, the fabrication of nanostructure with high surface area and porosity reduces the ion diffusion path length and multiplies the rate capability of the fabricated device [30, 91]. In the fabrication of supercapattery devices, use of gel electrolyte and binder free growth of active material on the current collector is also very fruitful to uplift the cyclic stability of the device [92].

The selection of highly capacitive electroactive material for supercapattery is pivotal, and it must comply with certain desirable characteristics. A highly polarizable capacitor-like electrode, i.e., having a wide potential window and a battery-like electrode combination, is much desirable for supercapattery devices. In such a device, the capacitor-like electrodes undergo the EDLC and/or pseudocapacitance, and the battery-like electrode offers redox or non-capacitive Faradaic reaction [43, 93] for improving the energy and power densities, respectively.

Ideally, the nanostructured carbon materials such as activated carbon (AC), carbon aerogel, carbon nanotubes (CNT) and graphene are the fundamental materials of selection for the polarizable negative electrode due to their higher surface area, chemical, thermal stability, micro/nanoporous structure and better electrical conductivity [94]. In addition to that, pseudocapacitive materials including metal oxides (i.e., RuO_2_, MnO_2_, Fe_2_O_3_, V_2_O_5_) and the conducting polymers (PANI, polypyrrole, PEDOT-PSS) can be used as the pseudocapacitive electrode materials, which provide a higher capacitance, but relatively narrow potential windows than EDLC materials. Mostly, the battery-type materials are used as positive electrode for supercapattery application and such materials are Co_3_O_4_, NiO and its ternary composition [95], etc.

The proper understanding of mechanism and progression of supercapattery devices can be accomplished through initial survey on the electrochemical performance of its constituents, i.e., capacitive- and Faradaic-type electrode materials for supercapattery. This preliminary study is truly obligatory as it can shed a light upon the contribution of these electrode materials in the device efficiency along with the effect of distinct charge storage mechanism on device performance. Eventually, the major goal of this overall discussion will be achieved through the review on the performance of various supercapattery devices.

## Non-Faradaic Capacitive (EDLC Type) Electrode Materials

The first supercapacitor was patented by General Electric (GE) in the year of 1957; then after SOHIO presented the first electric double-layer capacitor (EDLC) using carbon material. The carbon-based materials have attracted more interest in energy storage application due to their higher surface area, porous structure that allows the electrolyte ions to form an electrostatic double layer at the electrode/electrolyte interfaces and porous structure allowing electrolyte ions to move easily over the whole surface area of electrode materials. Moreover, carbon materials are of low cost, abundancy, appreciably high electrical conductivity and chemical stability. Further, they have controllable porosity, ease of handling and availability in various forms particularly, powders, sheets, fibers, aerogels, composites, foams, tubes, monoliths and nanohorns. [80, 96–98]. The allotropes of carbon materials such as graphene, carbon nanotube, graphite, activated carbon, fullerenes and amorphous carbon are used as electrode materials in EDLC supercapacitor. The specific capacitance of these electrodes are highly dependent on their surface area, pore size, surface functionality and electrical conductivity [99, 100]. Moreover, these materials are not only restricted as an electroactive material but also used a conductive binder, conducting network and current collector in the other type of supercapacitors. Mostly, carbon-based materials are used as a negative electrode for both symmetric, asymmetric and supercapattery devices. There are many reports published on the carbon-based materials for supercapacitor applications. In this section, we have highlighted the few breakthroughs in carbon-based electrode materials and summarized in Table 2.

**Table 2:** Summary of electrochemical performance of non-Faradaic capacitive electrode materials for supercapatteries

Sl. no.	Electrode	Scan rate/current density	Electrolyte	Specific capacitance	Potential window	Energy density (max)	Power density (max)	Cyclic stability	Refs.
1.	Ni(OH)_2_/AC/CNT║AC	0.5 A g^−1^	6 M KOH	82.1 F g^−1^	1.6 V	32.3 Wh kg^−1^	504.8 W kg^−1^	83.5% after 1000 cycles	[101]
2.	NiO–CNT║AC	2 mV s^−1^	1 M Na_2_SO_4_	197.7 F g^−1^	1.8 V	85.7 Wh kg^−1^	11.2 kW kg^−1^	91% after 4000 cycles	[102]
3.	CuCo_2_O_4_/CuO║AC	1 mV s^−1^	1 M KOH	141 F g^−1^	1.6 V	18 Wh kg^−1^	259 W kg^−1^	100% of after 5000 cycles	[103]
4.	Ni_2_P║BDAC	10 mV s^−1^	1 M KOH	121 F g^−1^	1.6 V	42 Wh kg^−1^	2856 W kg^−1^	100% of after 10,000 cycles	[110]
5.	NiCo_2_O_4_ nanoneedle║Walnut shells derived activated carbon	0.5 A g^−1^	2 M KOH	52.3 F g^−1^	1.7 V	21 Wh kg^−1^	424.5 W kg^−1^	99.3% after 5000 cycles	[114]
6.	Nickel cobalt sulfide@CNT║VN@CNT	0.4 A cm^−3^	PVA/KOH + LiCl/PVA	2332 F cm^−3^	1.6 V	30.64 mWh cm^−3^	80 mW cm^−3^	91.9% after 5000 bending cycles	[117]
7.	MnO_2_║rGO	0.1 A g^−1^	1 M Na_2_SO_4_	160 F g^−1^	2.0 V	22.2 Wh kg^−1^	101 W kg^−1^	90% after 3000 cycles	[133]
8.	CNT@Ni(OH)_2_║3D graphene networks (3DGNs)	1 A g^−1^	1 M KOH	124 F g^−1^	1.6 V	44.0 Wh kg^−1^	800 W kg^−1^	83% after 8000 cycles	[134]
9.	Lamellar holey graphene hydrogel (LGH)║BDTD-rGO	1 A g^−1^	1 M H_2_SO_4_	54 F g^−1^	1.4 V	9.52 Wh kg^−1^	0.45 kW kg^−1^	81.3% after 5000 cycles	[135]
10.	N doped reduced graphene oxide decorated on NiSe_2_║AC	1 A g^−1^	3 M KOH	113.8 F g^−1^	1.6 V	40.5 Wh kg^−1^	841.5 W kg^−1^	85.1% after 10,000 cycles	[136]
11.	NiCoP/NiCo-OH30║ZIF-67 (MOF) derived porous carbon	1 A g^−1^	6 M KOH	101 F g^−1^	1.6 V	34 Wh kg^−1^	11.6 kW kg^−1^	92% after 1000 cycles	[138]
12.	K_0.5_Mn_2_O_4_ nanosheets were subsequently grown on the carbon surface (NCMO)║ZIF-67 (MOF) derived nanoporous carbon	10 A g^−1^	1 M Na_2_SO_4_	75 F g^−1^	2.4 V	60 Wh kg^−1^	12.3 kW kg^−1^	92.6% after 10,000 cycles	[139]
13.	TM-nanorods║MOF derived N-doped hierarchical porous carbon nanorods (TM-NPs)	1 A g^−1^	2 M KOH	161 F g^−1^	1.5 V	47.1 Wh kg^−1^	17.1 kW kg^−1^	83.2% after 10,000 cycles	[140]
14.	MOF derived Mn_2_O_3_/C composite║AC	10 A g^−1^	1 M Na_2_SO_4_	45 F g^−1^	1.8 V	54.9 Wh kg^−1^	22.6 kW kg^−1^	97% after 5000 cycles	[141]

### Activated Carbon

The activated carbon (AC) is one type of carbon materials, which is used as conductive agent in most of the energy storage devices because of its comparably good electrical conductivity, porous structure, cost effectiveness and ease of availability of material. Similar way, AC was used as a positive/negative electrode for supercapacitor devices and carbon backbone for growing a hierarchical nanostructure. Further, it can be used to make a composite with metal oxides and with conducting polymers, to improve the energy density of the composite materials through Faradaic reaction and can provide a good conducting path for electrons. Beside this, the functionalization of carbon materials is also another way to enhance the energy storage capacity by improved surface wettability of active materials, which allows to penetrate the electrolyte through porous structure resulting to a higher electrostatic double layer formation. Here, we have summarized some of the important results, where activated carbon is used as a negative electrode for supercapacitor applications.

Sui et al. [101] synthesized Ni(OH)_2_/AC/CNT composite by microwave-assisted method for asymmetric supercapacitor device. Further, this group constructed the device using Ni(OH)_2_/AC/CNT as a positive electrode and AC as a negative electrode. The fabricated device displayed a specific capacitance of 82.1 F g^−1^ with a wide operating potential widow of 1.6 V. Finally, the device showed the energy density of 32.3 Wh kg^−1^ at a power density of 504.8 W kg^−1^ with 83.5% capacitance retention after 1000 cycles. Roy et al. [102] established an asymmetric supercapacitor device with high energy density of 85.7 Wh kg^−1^ at a power density of 11.2 kW kg^−1^ by employing the NiO–CNT as a positive and AC as a negative electrode, respectively.

Shanmugavani and Selvan [103] constructed an asymmetric supercapacitor using AC as a negative electrode and CuCo_2_O_4_/CuO nanocomposites as a positive electrode, and it showed an energy density of 18 Wh kg^−1^ at a power density of 259 W kg^−1^ with 100% of capacitance retention after 5000 cycles. Likewise, a various hybrid supercapacitor devices are fabricated using AC as a negative electrode, e.g., AC║Co_2_MnO_4_/Co [104], AC║MnO_2_ [105], AC║Co(OH)_2_/Ni foam [106] and AC║Ni(OH)_2_ [107] and those devices exhibited the energy density in the range of 15–40 Wh kg^−1^ [101].

**Fig. 4:**
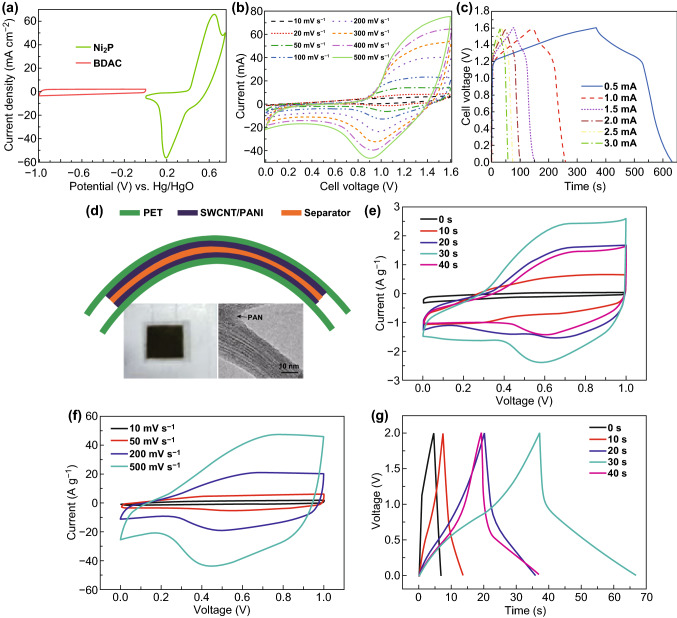
**a** CV curve for Ni_2_P and BDAC at 5 mV s^−1^. **b** CV and **c** galvanostatic charge/discharge curves for asymmetric supercapacitors (Ni_2_P║BDAC). Reprinted with permission from Ref. [110]. Copyright 2018 American Chemical Society. **d** Schematic representation of the SWCNT/PANI symmetric device. Inset is the digital image of the fabricated device and TEM image of SWCNT/PANI. **e** CVs of SWCNT/PANI at various PANI deposition time. **f** CV profiles of SWCNT/PANI device versus scan rates. **g** Galvanostatic charge/discharge curves of SWCNT/PANI at different PANI deposition time. Adapted with permission from [119]. Copyright 2012 The Royal Society of Chemistry

Recently, biomass-derived carbon gained more interest in the energy storage applications because of low cost, simple preparation process and eco-friendliness. Various bio-wastes are available in different forms, which can be turned into porous carbon materials for the EDLC application. Liu et al. [108] derived the activated carbon (silica less) from rice husk for supercapacitor electrode, and it has obtained the specific capacitance of 278 F g^−1^ at a current density of 0.5 A g^−1^. The activated carbon from carbonaceous mudstone and lignin delivered the specific capacitance of 155.6 F g^−1^ [109]. Surendran et al. [110] prepared a flexible supercapattery gadget using the biomass-derived activated carbon (BDAC) as a negative electrode and nickel phosphide (Ni_2_P) as a positive electrode, which delivered an energy density of 42 Wh kg^−1^ and power density of 2856 W kg^−1^. Figure 4a shows the CV curves of the positive and negative electrodes of Ni_2_P and BDAC at a scan rate of 5 mV s^−1^ in three electrode system and CV (Fig. 4b), GCD (Fig. 4c) curves of supercapattery at different scan rates and currents. Wei et al. [111] prepared the high surface area activated carbon (2100–2970 m^2^ g^−1^) for supercapacitor electrode from various types of bio-waste such as potato starch, cellulose and eucalyptus wood sawdust, which delivered a specific capacitance of 140–240 F g^−1^ in organic electrolyte. Schlee et al. [112] assembled a free-standing supercapacitor electrode using the kraft lignin-based bio-waste. The kraft lignin-derived carbon electrode shows an output of specific capacitance 155 F g^−1^ at current density of 0.1 A g^−1^ with good capacitance retention (94%) after 6000 cycles. Martínez-casillas et al. [113] utilized pecan nutshell bio-waste to produce activated carbon, and this electrode delivered a specific capacitance of 150 F g^−1^ at low scan rate (5 mV s^−1^). Wang et al. [114] engineered an asymmetric supercapacitor device using walnut shell-derived activated carbon and NiCo_2_O_4_ nanoneedle electrodes. This device acquired energy density of 21 Wh kg^−1^ at a power density of 424.5 W kg^−1^ along with 99.3% capacitance retention after 5000 cycles at a current density of 4 A g^−1^.

### Carbon Nanotubes

Carbon nanotubes (CNTs) are one of the promising materials for electrochemical energy storage applications due to their higher electrical conductivity because of one-dimensional structure and higher active surface area [115, 116]. The CNT electrode showed a higher specific capacitance of ~ 100–400 F g^−1^, which is better than conventional carbon-based electrodes due to its high electrical conductivity, fast charge transportation, higher surface-to-volume ratio and tremendous electrolyte accessibility [115]. Similar to activated carbon materials, CNT is also hybridized with metal oxides (e.g., manganese oxide) or conductive polymers (e.g., polyaniline) for improving the energy density. Liu et al. [117] reported the in-situ growth of vertically aligned nickel–cobalt sulfide nanowires on CNT for fiber-based asymmetric supercapacitor application. The as-prepared electrode achieved a volumetric capacitance of 2332 F cm^−3^, and the device delivered a high volumetric energy density of 30.64 mWh cm^−3^.

Lee et al. [118] reported the oxidized carbon nanotube as an electrode material for this application, which displayed an exceptionally higher gravimetric energy (~ 200 Wh kg^−1^) and power density (~ 10 kW kg^−1^). Xiao et al. [90] synthesized a free-standing mesoporous vanadium nitride/CNT-based hybrid electrode material and applied it for flexible supercapacitor application. The fabricated flexible supercapacitor showed a higher volumetric capacitance of 7.9 F cm^−3^ and a power density of 0.4 W cm^−3^. Similarly, the CNT/PANI free-standing electrode (Fig. 4d–g) delivered a high energy and power density values of 131 Wh kg^−1^ and 62.5 kW kg^−1^, respectively [119]. Further, Adusei et al. [120] reported the functionalized CNT by oxygen plasma method and it showed a higher cycle stability of 93.2% after 4000 cycles.

### Carbon Onion

**Fig. 5:**
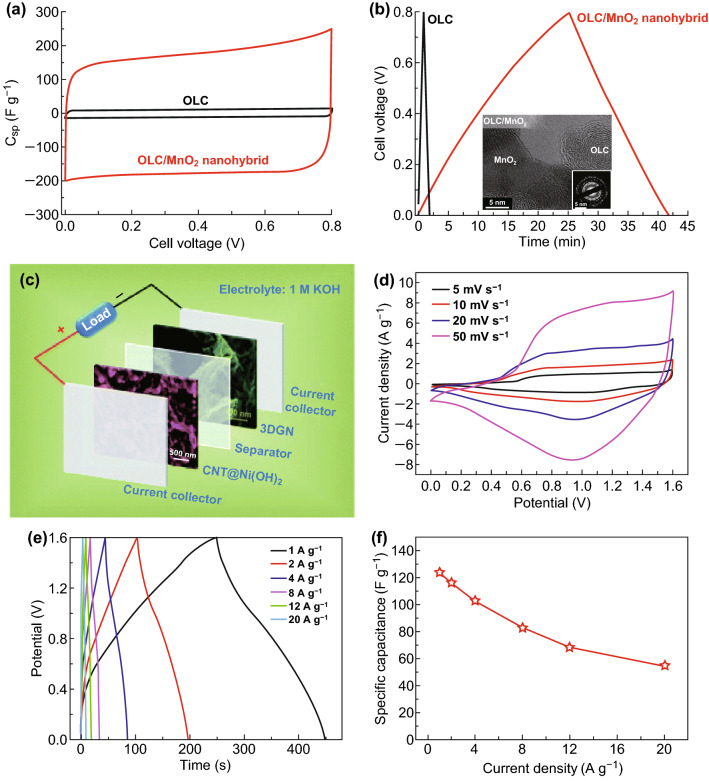
**a** CV and **b** galvanostatic charge/discharge curves of onion-like carbon (OLC) and OLC/MnO_2_ symmetric cell at 5 mV s^−1^. The inset is the TEM and SAED pattern of OLC/MnO_2_. Reproduced with permission from Ref. [124]. Copyright 2015 The Royal Society of Chemistry. **c** Schematic illustration of CNT@Ni(OH)_2_║3DGN asymmetric device, **d** CV and **e** galvanostatic charge–discharge profiles of CNT@Ni(OH)_2_║3DGN asymmetric device. **f** Specific capacitance versus current densities. Adapted with permission from Ref. [134]. Copyright 2015 The Royal Society of Chemistry

Carbon onion is another special material in the carbon family other than CNTs, graphene; because of its zero dimension with concentric shell structure, provides higher active area for the double-layer capacitance [68]. Further, it has higher inter-particle pore volume, a high accessible outer surface area with higher electrical conductivity but lacks of energy density. Carbon onion is perfectly fit as an electrode material for energy storage device when implemented to redox active materials like surface functional groups, molecular species (e.g., quinones). Among the materials like manganese oxide, ruthenium oxide, nickel hydroxide, PANI, polypyrrole (PPy), etc, the manganese oxide/carbon onion composite showed the maximum specific capacitance of 575 F g^−1^ in 0.5 M H_2_SO_4_ electrolyte [121]. Borgohain et al. [122] reported the polydiallyldimethylammonium chloride (PDDA) modified carbon nano-onion with MnO_2_ (55 wt%) symmetric cell, which delivered a maximum capacitance of 218 F g^−1^ in liquid electrolyte with a high energy density of 6.14 Wh kg^−1^. Wang et al. [123] reported the (MnO_2_)/onion-like carbon (OLC)-based nanocomposite electrode with the specific capacitance of 177.5 F g^−1^. Also, Makgopa et al. [124] synthesized nano-diamond-derived carbon onion with MnO_2_ composite electrodes (Fig. 5a, b) and it showed the specific capacitance of 335–408 F g^−1^ at current densities of 0.1–0.3 A g^−1^. Further, Mykhailiv et al. [125] reported the functionalization of carbon nano-onions (CNOs) with sodium dodecyl sulfate (SDS) and polypyrrole (PPy). Finally, the functionalized CNOs/SDS/PPy electrode delivered an extremely high capacitance of 800 F g^−1^; likewise, the bilayered CNOs/PPy delivered the specific capacitance of 1300 F g^−1^. Also, Plonska-Brzezinska [126] reported the functionalized carbon nano-onions (CNOs/4-ABAc/PANI) electrode, which exhibited a specific capacitance of 206.6 F g^−1^. Jin et al. [127] fabricated rice-husk extracted porous carbon nano-onions with high specific capacitance of 350 F g^−1^ with good capacitance retention of 99% even after 10,000 cycles.

### Graphene

Graphene is another allotrope of carbon material, which has one atom thin-layered material with *sp*^2^ hybridized carbon lattice. Graphene is a two-dimensional layered structure with extraordinary physico-chemical properties like high surface area, higher electrical, mechanical properties and electrochemical performance and so on [128–131]. Due to its extraordinary properties, it is highly utilized for different energy conversion and storage applications. Various methods are adopted for the synthesis of graphene or reduced graphene oxides such as chemical, thermal and hydrothermal methods. Zhang et al. [132] reported a biomass derived high surface area porous 3D graphene (3523 m^2^ g^−1^) with specific capacitance of 231 F g^−1^ and energy density of 98 Wh kg^−1^. Similar to other carbon allotropes, graphene also composite with various materials for improving the energy density. Shivakumar and Munichandraiah [133] reported an MnO_2_║rGO asymmetric supercapacitor with energy density of 22.2 Wh kg^−1^ and power density of 101 W kg^−1^. Yi et al. [134] reported CNT@Ni(OH)_2_║3D graphene asymmetric supercapacitor, which delivered an energy density of 44.0 Wh kg^−1^ and power density of 800 W kg^−1^ by synergic effect of both electrodes. Figure 5c–f shows the schematic diagram, CV curves, GCD curves and specific capacitances of the asymmetric device, respectively. With increasing scan rate, the performance of the asymmetric device keeps its ideal characteristics, which indicates the better capacitive behavior. Li et al. [135] reported functionalized 3D graphene xerogel (BDTD-rGO)-based supercapacitor with specific capacitance of 360 F g^−1^ with superior cyclic stability of 96.4% after 10,000 cycles. Gu et al. [136] reported the NiSe_2_ decorated nitrogen doped reduced graphene oxide supercapattery device with maximum energy density of 40.5 Wh kg^−1^ and power density of 841.5 W kg^−1^.

### Metal Organic Framework-Derived Carbon Electrode Materials

As advanced electrode material for electrochemical energy storage application, researchers discovered novel metal organic frameworks (MOFs). In general, pristine MOFs are being used as positive electrodes for supercapattery device, whereas MOF-derived carbon as negative one. Compared to pure carbon-based materials, these MOF-derived carbon materials deliver excellent electrochemical performance owing to their favorable natures like high porosity, high specific surface area, etc. [137]. Annealing of MOFs at high temperature under inert atmosphere converts them into carbon, retaining the original MOF template. Implementing a zinc-based MOF, i.e., zeolitic imidazole framework (ZIF-8), Javed et al. [137] fabricated a nanoporous carbon derived from zinc-based MOF as negative electrode material for electrochemical supercapacitor application. Similarly, Li et al. [138] synthesized a ZIF-67 (Co-based zeolitic imidazole framework)-derived porous carbon (PC) which obtained gravimetric specific capacitance of 150 F g^−1^ at current density of 1 A g^−1^. Wei et al. [139] prepared a ZIF-67-derived nanoporous carbon (NC) for supercapacitor applications. The prepared electrode delivered a specific capacitance of 269 F g^−1^ at a current density of 1 A g^−1^ along with 90.4% capacitance retention even after 10,000 cycles. So, depending upon the selection of different metal ions, their corresponding MOFs can provide carbon material with better electrochemical efficiency. Nitrogen-doped MOF-derived carbon material can provide even higher performance due to the added wettability and conductivity by nitrogen. Hence, Qu et al. [140] prepared N-doped hierarchical porous carbon nanorods (TM-NPs) by annealing Ni-based MOF at 1000 °C for 3 h under an argon atmosphere. The obtained specific capacitance of this electrode was 330 F g^−1^ at current density of 1 A g^−1^. Instead of converting, the MOF material completely into carbon material if the MOF is first converted to metal oxide and over that carbon is coated then that can be used as excellent carbon-based material for supercapattery application with enhanced performance. Nagamuthu et al. [141] synthesized a MOF-derived Mn_2_O_3_/C composite electrode, which showed the specific capacitance of 776 F g^−1^ at a current density of 1 A g^−1^. The higher capacitance due to the presence of carbon in the composite, which provides good electrical conductivity during the electrochemical reaction. There are various strategies being followed to improve the energy density of the carbon materials, but the performance of the carbon materials is below the expected values, so further research is required to improve the performance of this material to make it as a good candidate for supercapattery applications.

## Faradaic (Pseudocapacitive and Battery Type) Electrode Materials

**Fig. 6:**
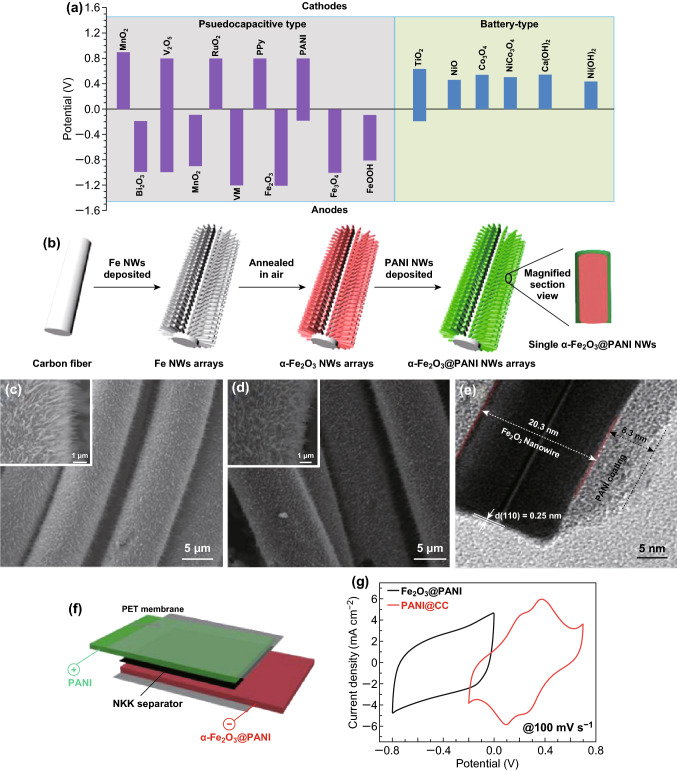
**a** The potential window of the various redox active materials (pseudocapacitive and battery type) in aqueous electrolyte. Reproduced with permission from Ref. [77]. Copyright 2015 The Royal Society of Chemistry. **b** Schematic representation of electrode fabrication of α-Fe_2_O_3_@PANI nanowires. FE-SEM images of **c** α-Fe_2_O_3_ and **d** α-Fe_2_O_3_@PANI **e** TEM image of α-Fe_2_O_3_@PANI. **f** Schematic representation of asymmetric supercapacitors and **g** CV curves of PANI@CC and α-Fe_2_O_3_@PANI electrodes at 100 mV s^−1^. Reproduced with permission from Ref. [165]. Copyright 2015 American Chemical Society

The positive electrode of supercapattery is usually made up of high-energy battery-type or pseudocapacitive materials and its composite. The energy density of the electrode mainly depends on the capacitance of the electrode, so utilization of its full theoretical capacitance of the materials is critical. Therefore, it is essential to study the various parameters, which are directly related to the capacitance of the material. The battery electrodes store the charges through reversible Faradaic reaction, and it mainly depends on surface area, short diffusion path for electron and ions, higher electrical conductivity with multiple oxidation states. The Faradaic reaction occurs in both pseudocapacitor and battery electrode but the electrochemical behavior like CV, GCD are different due to phase change in battery-type material. Transition metal oxides and conducting polymer electrode materials are coming under the category of pseudocapacitance; in that some of the transition metal oxides such as Co-, Ni-based oxides are used as battery-type electrodes, because of their multivalent oxidation states available for charge storage. Figure 6a summarizes the operational potential window of various materials (pseudocapacitive and battery-type) including the positive and negative electrodes in water-based aqueous electrolyte [77]. Table 3 summarizes the electrochemical performance of Faradaic electrode materials including positive and negative electrodes.

**Table 3:** Summary of electrochemical performance of Faradaic electrode materials for supercapatteries

Sl. no.	Electrode	Scan rate/current density	Electrolyte	Specific capacitance	Potential window	Energy density (max)	Power density (max)	Cyclic stability	Refs.
1.	Fe_2_O_3_@ACC║Fe_2_O_3_@ACC	1 mA cm^−2^	2 M LiCl	1565 mF cm^−2^	1.8 V	9.2 mWh cm^−3^	12 mW cm^−3^	95% after 4000 cycles	[88]
2.	RuO_2_/Co(OH)_2_║AC	1.5 A g^−1^	2 M KOH	164 F g^−1^	0.7 V	58.4 Wh kg^−1^	1.2 kW kg^−1^	86% after 5000 cycles	[144]
3.	MnO_2_ nanocomposite║AC	NA	[Bmim]PF_6_/DMF	NA	3 V	67.5 Wh kg^−1^	20.4 KW kg^−1^	NA	[148]
4.	CNT–MnO_2_ nanocomposite film║FeSe_2_ nanonuts on carbon fiber	0.698 A g^−1^	LiCl gel	77.83 F g^−1^	1.7 V	27.14 Wh kg^−1^	571.3 W kg^−1^	80.3% after 8000 cycles	[150]
5.	Ni(OH)_2_ thin films║AC	0.9 A g^−1^	6 M KOH	192 F g^−1^	1.6 V	68 Wh kg^−1^	44 kW kg^−1^	~90% after 10,000 cycles	[153]
6.	NiO nanocubes║AC	1 mA cm^−2^	6 M KOH	42 mF cm^−2^	1.5 V	0.066 mWh cm^−3^	3.796 W cm^−3^	89.3% after 10,000 cycles	[156]
7.	NiO nanotube array║Fe_2_O_3_	5 A g^−1^	6 M KOH	103.275 F g^−1^	1.7 V	48 Wh kg^−1^	2089 W kg^−1^	85.2% after 5000 cycles	[158]
8.	Polyaniline║α-Fe_2_O_3_/polyaniline	1 A g^−1^	PVA/LiCl gel	112.6 F g^−1^	1.8 V	0.31 mWh cm^−3^	67.1 mW cm^−3^	80.3% after 5000 cycles	[162]
9.	Cobalt oxide║iron oxide	0.5 A g^−1^	6 M KOH	94.5 F g^−1^	1.6 V	40.53 Wh kg^−1^	2432 W kg^−1^	75% after 12,000 cycles	[163]
10.	MnO_2_ nanowire║Fe_2_O_3_ nanotube	2 mA cm^−2^	1 M H_2_SO_4_	91.3 F g^−1^	1.6 V	0.55 mWh cm^−3^	NA	NA	[164]
11.	α-Fe_2_O_3_@PANI core–shell nanowire║PANI nanorods/CC (carbon cloth)	5 mV s^−1^	1 M Na_2_SO_4_	2.02 mF cm^−3^	1.5 V	0.35 mWh cm^−3^	120.51 mW cm^−3^	95.77% after 10,000 cycles	[165]
12.	MnO_2_@TiN║N-MoO_3-x_	0.25 mA cm^−1^	PVA/LiCl gel	4.1 F cm^−3^	2.0 V	2.29 mWh cm^−3^	0.76 µW cm^−3^	NA	[169]
13.	Ni,Co–OH/rGO║3D hierarchical porous carbon	0.1 A g^−1^	6 M KOH	162 F g^−1^	1.6 V	56.1 Wh kg^−1^	76 W kg^−1^	80% after 17,000 cycles	[170]
14.	α-MnO_2_@NiCo_2_O_4_core-shell heterostructures║3D N-doped porous nanocage carbon	1 A g^−1^	PAAK/KOH gel	110.6 F g^−1^	1.7 V	46.2 Wh kg^−1^	15.3 kW kg^−1^	90% after 2000 cycles	[171]
15.	Ferric–cobalt–nickel ternary oxide nanowire arrays/graphene fibers║polyaniline-derived carbon nanorods/graphene fibers	0.1 mA cm^−2^	PVA/KOH	61.58 mF cm^−2^	1.4 V	16.76 µWh cm^−2^	69.94 μW·cm^−2^	90.9% after 4000 cycles	[172]
16.	NiCoMn ternary metal oxide flakes on reduced graphene oxide (NCMO/rGO)║rGO	0.5 A g^−1^	3 M KOH	207.36 F g^−1^	1.6 V	27 Wh kg^−1^	0.598 kW kg^−1^	96% after 2000 cycles	[173]
17.	Mn–Ni–Co ternary oxides/rGO║rGO	NA	6 M KOH	NA	1.4 V	35.6 Wh kg^−1^	699.9 W kg^−1^	77.2% after 10,000 cycles	[174]
18.	NiCo_2_O_4_@GQDs║AC	4 A g^−1^	2 M KOH	95 F g^−1^	1.6 V	38 Wh kg^−1^	800 W kg^−1^	71.8% after 3000 cycles	[175]
19.	2D copper cobalt oxide (CCO-NS)║HCP-CNF	1 A g^−1^	3 M KOH	244 F g^−1^	1.6 V	25.1 Wh kg^−1^	400 W kg^−1^	91.1% after 5000 cycles	[176]
20.	Ni-MOF║AC	0.5 A g^−1^	3 M KOH	87 F g^−1^	1.4 V	21.05 Wh kg^−1^	6.03 kW kg^−1^	70% after 2000 cycles	[182]
21.	3D co-doped Ni-based MOF║AC	NA	6 M KOH	NA	1.5 V	25.92 Wh kg^−1^	NA	78.1% after 6000 cycles	[183]
22.	(Ni-MOF derived) NiO║AC	0.5 A g^−1^	2 M KOH	108 F g^−1^	1.6 V	38.4 Wh kg^−1^	0.74 kW kg^−1^	82% after 5000 cycles	[184]
23.	ZCCO║rGO	NA	3 M KOH	NA	1.6 V	38.4 Wh kg^−1^	16 kW kg^−1^	94% after 5000 cycles	[185]
24.	NiCo-MOF║AC	0.5 A g^−1^	PBI-KOH	172.7 F g^−1^	1.8 V	77.7 Wh kg^−1^	0.45 kW kg^−1^	92.7% after 4000 cycles	[187]
25.	NiCo-MOF/AB║AC	0.5 A g^−1^	2 M KOH	115.05 F g^−1^	1.5 V	33.84 Wh kg^−1^	15.1 kW kg^−1^	84.2% after 5000 cycles	[188]
26.	Ni-MOF@CNT/GN║AC	0.1 mA cm^−2^	PVA-KOH	898 mF cm^−2^	1.65 V	135.84 Wh kg^−1^	NA	93% after 4000 cycles	[189]
27.	NiCo-MOF@PNT║AC	0.5 A g^−1^	1 M KOH	132 F g^−1^	1.5 V	41.2 Wh kg^−1^	NA	79.1% after 10,000 cycles	[190]
28.	CNT–CuCo_2_O_4_@Ag║AC	NA	PVA-KOH	NA	1.6 V	91 Wh kg^−1^	18 kW kg^−1^	98% after 20,000 cycles	[191]
29.	Modified MXene (2D Ti_3_C_2_)║Modified MXene (2D Ti_3_C_2_)	1 A g^−1^	1 M H_2_SO_4_ +1 M Li_2_SO_4_	NA	1.6 V	27.4 Wh kg^−1^	4 kW kg^−1^	90.4% after 5000 cycles	[197]

### Transition Metal Oxides

For pseudocapacitor applications, mostly RuO_2_-based electrode material is used due to their good proton conductivity, high specific capacitance, high rate capacity, wide potential window (up to 1.2 V), higher reversible surface redox reactions and long cycle life. The nanotubular arrayed hydrous RuO_2_ electrode showed a maximum specific capacitance of 1300 F g^−1^ with good cycle stability [142]. Even though it has higher specific capacitance and good cycle stability, the real-time application is challenging due to its higher cost and toxicity to the environment. Edison et al. [143] reported the carbon encapsulated RuO_2_ nanorods (RuO_2_ NRs/C) electrode with specific capacitance of 151.3 F g^−1^. Li et al. [144] fabricated and reported the asymmetric supercapacitor of hydrous RuO_2_@ Co(OH)_2_║AC with energy and power densities of 58.4 Wh kg^−1^ and 1.2 kW kg^−1^, respectively. Similar to RuO_2_, MnO_2_ is also considered as a good material for pseudocapacitor, due to low cost, abundance, environmental friendliness and the high theoretical capacitance (1380 F g^−1^). However, the achievable capacitance of these materials is ~ 200 F g^−1^ due to its poor electrical conductivity [145]. So, it is important to improve the electrical conductivity of MnO_x_ material as well as other metal oxides by hybridizing with carbon materials. The hybridization of MnO_2_ nanosheets with CNT improves the specific capacitance to 325.5 F g^−1^ because of the higher surface area (127 m^2^ g^−1^) and improved conductivity by CNT [146]. The specific capacitance further increased by introducing the nickel foam substrate to hybridize MnO_2_/CNT electrode material. By this way, Chen et al. [147] reported a manganese dioxide/multiwall carbon nanotube/Ni foam (MnO_2_/MWNT/Ni foam) electrode with high specific capacitance of 355.1 F g^−1^. Similarly, Zhang et al. [148] constructed an MnO_2_ nanocomposite║AC asymmetric supercapacitor in ionic liquid electrolyte. The fabricated asymmetric device exhibited a specific capacitance of 523.3 F g^−1^ with the wider operating potential of 2.1 V. Xia et al. [149] reported a porous δ-MnO_2_ electrode with higher specific capacitance of 411 F g^−1^ at a scan rate of 5 mV s^−1^. Wang et al. [150] reported a flexible fiber-based CNT–MnO_2_║FeSe_2_ nanonuts asymmetric supercapacitor with maximum energy density of 27.14 Wh kg^−1^ at a power density of 571.3 W kg^−1^. Toupin et al. [151] reported the maximum specific capacitance of 1380 F g^−1^ for MnO_2_ thin film-based electrode in three-electrode system.

Compared to the pseudocapacitive material, battery-type material possesses a higher theoretical capacitance due to their good intercalation based redox activity and multivalent oxidation states. Nickel oxide (NiO) is one of the battery-type materials with a very high theoretical capacitance of 3750 F g^−1^ [152–154]. Similarly, its hydroxides and its ternary composite also received more attention due to its higher theoretical capacitance and ease to grow various nanostructures on different substrates; moreover, it has higher inherent electrical conductivity. The various form of nickel oxide-based electrode material was used in this application. The mesoporous Ni(OH)_2_ nanoflakes electrode achieved a specific capacitance of 2055 F g^−1^ [155], NiO nanocubes with areal capacitance of 1012 mF cm^−2^ [156], Ni(OH)_2_/UGF (ultra-thin graphene foam) asymmetric supercapacitor with very high power density of 44 kW kg^−1^ [157]. Similarly, Lin et al. [158] fabricated asymmetric supercapacitor with NiO nanotube array and Fe_2_O_3_ electrodes on carbon paper and this device obtained a high energy density of 48 Wh kg^−1^ at a power density of 2089 W kg^−1^.

The iron oxide is one of the transition metal oxides, highly explored for energy storage application as a negative electrode, due to its earth abundance, low cost, multiple oxidation states and high theoretical specific capacitance [159]. Li et al. [160] reported a hydrothermally synthesized α-Fe_2_O_3_ nanosheets electrode, which delivered a specific capacitance of 279.9 F g^−1^ at a scan rate of 5 mV s^−1^. Binitha et al. [161] reported a porous α-Fe_2_O_3_ fiber electrode with a specific capacitance of 256 F g^−1^. Li et al. [88] fabricated a porous Fe_2_O_3_ nanospheres assembled on activated carbon cloth exhibiting superior areal capacitance of 2775 mF cm^−2^. Yang et al. [162] prepared hierarchical nanostructured α-Fe_2_O_3_/polyaniline achieving a high specific capacitance of 473.6 F g^−1^ at a current density of 1 A g^−1^ along with 98.2% capacitance retention after 5000 cycles. Likewise, a various nanostructure of iron oxide is used as a negative electrode for an asymmetric device. For example, Pai and Kalra [163] reported an aqueous electrolyte-based Co_3_O_4_║Fe_2_O_3_ asymmetric supercapacitor with a specific capacitance of 94.5 F g^−1^; energy and power densities are 40.53 Wh kg^−1^ and 2432 W kg^−1^, respectively. Similarly, Yang et al. [164] fabricated a flexible MnO_2_ nanorod║Fe_2_O_3_ nanotube asymmetric supercapacitor, which delivered a higher volumetric capacitance of 0.55 mWh cm^−3^. Lu et al. [165] constructed an asymmetric supercapacitor using α-Fe_2_O_3_@PANI core–shell nanowire arrays as anode and PANI nanorods on carbon cloth as a cathode. The α-Fe_2_O_3_@PANI core–shell nanowire arrays are fabricated by a facile and cost-effective electrodeposition method. The detailed fabrication process is schematically provided in Fig. 6b, c, d indicating the SEM images of α-Fe_2_O_3_ nanowire arrays before and after PANI coating. The result clearly shows the formation of highly dense, uniform growth of α-Fe_2_O_3_ nanowire on carbon fiber. Further, a thin uniform coating of PANI layer over α-Fe_2_O_3_ nanowire was observed from SEM as well as TEM images (Fig. 6e). Moreover, the asymmetric supercapacitor device was (Fig. 6f) fabricated using α-Fe_2_O_3_@PANI and PANI electrodes and it has delivered a high volumetric capacitance of 2.02 mF cm^−3^, a high energy density of 0.35 mWh cm^−3^ and a power density of 120.51 mW cm^−3^ with an excellent capacitance retention of 95.77% even after 10,000 cycles. Similar to other metal oxides, iron oxide is also facing the poor conductivity issue, which reduces its specific capacitance considerably; hence, it is necessary to hybridize iron oxide with carbon materials to improve the electrical conductivity. There are various composite-based electrodes developed for this application, such as graphene–Fe_2_O_3_ composite electrode with an improved specific energy density of 65–204 Wh kg^−1^ against Li^+^ ions intercalation. Yang et al. [166] reported a porous α-Fe_2_O_3_/graphene composite electrode with a higher specific capacitance of 343.7 F g^−1^ and a very high stability of 95.8% (capacitance retention) after 50,000 cycles. Interestingly, Ye et al. [167] fabricated a carbon coated ferric oxide nanoparticles (Fe_2_O_3_@C) electrode, which delivered an exceptionally higher specific capacitance of 612 F g^−1^ with an excellent capacitance retention of 90% after 10,000 cycles. On the other hand, molybdenum trioxide is also an excellent electrode material for supercapacitor applications due to its exceptionally high work function (6.9 eV), low cost and variable oxidation states of Mo [168]. Yu et al. [169] reported the N-doped molybdenum trioxide nanowires (N-MoO_3-x_) as anode material for a flexible fiber-shaped asymmetric supercapacitor, which delivered the energy density of 2.29 mWh cm^−3^ and a power density of 0.76 µW cm^−3^.

Recently, ternary metal oxide-based materials are gaining great interest in energy storage applications due to their multivalent oxidation states of different metal ions and higher electrical conductivity compared to its binary counterpart. Ma et al. [170] reported an Ni,Co–OH║rGO asymmetric supercapacitor with a higher energy density of 56.1 Wh kg^−1^ and good capacitance retention of 80% even after 17,000 cycles. Ma et al. [171] reported an all-solid-state asymmetric supercapacitor with α-MnO_2_@NiCo_2_O_4_ core–shell heterostructures, and it delivered a maximum energy density of 46.2 Wh kg^−1^ at maximum power density of 15.3 kW kg^−1^. Likewise, Singh et al. [131] reported a ternary Co_3_O_4_–MnO_2_–NiO hybrid 1D nanotube array with a higher specific capacitance of 2525 F g^−1^ and good cycling stability of 80% after 5700 cycles. Zhao et al. [172] constructed a flexible fiber-based asymmetric supercapacitor based on hierarchical ferric–cobalt–nickel ternary oxide nanowire arrays and polyaniline-derived carbon nanorods on graphene fiber as positive and negative electrodes. The asymmetric device delivered a specific areal capacitance of 61.58 mF cm^−2^ with an energy density of 16.76 µWh cm^−2^. Sanchez et al. [173] fabricated an asymmetric device based on a novel porous NiCoMn ternary metal oxide flakes on reduced graphene oxide (NCMO/rGO) as a positive electrode and rGO as a negative electrode, and it has delivered a specific energy density of 27 Wh kg^−1^ with 96% capacitance retention after 2000 cycles. Furthermore, Wu et al. [174] fabricated and studied an Mn–Ni–Co ternary oxides/rGO and rGO-based asymmetric supercapacitor. The asymmetric device delivered a maximum energy density of 35.6 Wh kg^−1^ at a power density of 699.9 W kg^−1^ with 77.2% capacitance retention even after 10,000 cycles. Luo et al. [175] employed graphene quantum dots decorated NiCo_2_O_4_ (NiCo_2_O_4_@GQDs) as positive and AC as negative electrodes for asymmetric supercapacitor, which delivered the energy density of 38 Wh kg^−1^ at a power density of 800 W kg^−1^. Babu et al. [176] assembled an asymmetric supercapacitor using 2D copper cobalt oxide (CCO-NS) as positive and from hyper cross-linked polymers (HCP-CNF) as negative electrode delivering the energy density of 25.1 Wh kg^−1^ at a power density of 400 W kg^−1^ with maximum operating voltage 1.6 V and excellent capacitance retention 91.1% after 5000 cycles.

### MOF-Derived Metal Oxides

**Fig. 7:**
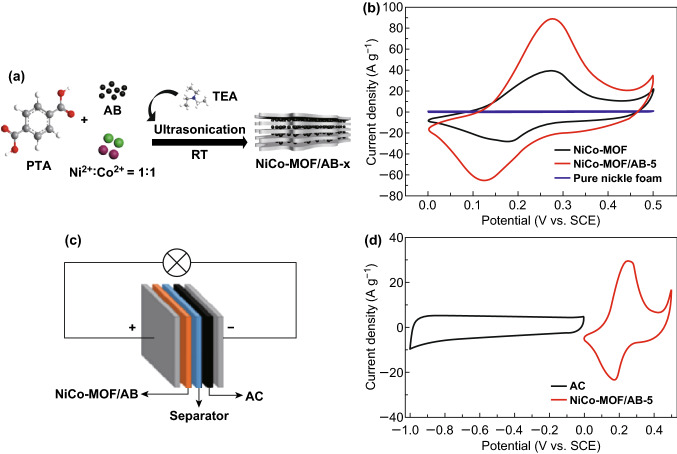
**a** Schematic illustration of synthesis of NiCo-MOF/AB composite. **b** Cyclic voltammetry (CV) curve of pure nickel foam, NiCo-MOF and NiCo-MOF/AB composite at a scan rate 50 mV s^−1^. **c** Schematic diagram of supercapattery device NiCo-MOF/AB║AC. **d** CV curves of NiCo-MOF/AB composite and AC. Adapted with permission from Ref. [188]. Copyright 2019 Elsevier

Metal organic frameworks (MOFs) are coordination polymers formed via the strong bonding between central metal ions and organic linkers [177]. Intrinsic properties like high porosity, surface area and appreciable aspect ratio of MOFs make them very useful for the application of supercapacitors [178–181]. Du et al. [182] synthesized a nickel-based MOF (Ni-MOF) with hierarchical porous nanosheets to utilize as positive electrode and activated carbon (AC) as negative electrode for supercapattery device. Appreciable potential window of 1.4 V was achieved by this device along with energy density of 21.05 Wh kg^−1^ and 70% cyclic stability with 2000 cycles. Poor electronic conductivity of pure MOFs is impeding them in obtaining good electrochemical performance. So, metal with good electrical conductivity can be doped with pristine MOF to get supercapattery performance. Wang et al. [183] fabricated a 3D Co-doped Ni-based MOF for positive electrode material and activated carbon (AC) as negative electrode for supercapattery device. Energy density of 25.92 Wh kg^−1^ and 78.1% cyclic stability with 6000 cycles was obtained by this device. Meng et al. [184] synthesized the NiO from a nickel-based MOF (Ni-MOF) for the positive electrode of supercapattery. The designed NiO║AC device obtained a energy density of 38.4 Wh kg^−1^ with 82% capacitance retention after 5000 cycles. Saleki et al. [185] fabricated a high-efficient supercapattery implementing nanoporous double-shelled CuCo_2_O_4_ (ZCCO) and reduced graphene oxide (rGO) for supercapattery device. The device ZCCO║rGO revealed maximum energy density 38.4 Wh kg^−1^ and maximum power density 16 kW kg^−1^ along with an excellent capacitance retention of 94% even after 5000 charge/discharge cycles. Instead of using a single metal-based MOF, the use of bimetallic MOF could enhance the electrochemical performance due the added electronic conductivity and redox active centers of two different metals [186]. Following this, Ye et al. [187] designed a supercapattery device out of NiCo-MOF and activated carbon (AC), i.e., NiCo-MOF║AC. The fabricated NiCo-MOF║AC device delivered the specific capacitance of 172.7 F g^−1^ at a current density of 0.5 A g^−1^ with very good cyclic stability of 92.7% even after 4000 cycles. The high electronic conductivity of carbon-based materials can be well exploited by making composites of them with bimetallic MOFs. Liu et al. [188] synthesized bimetallic MOF composite with acetylene black (AB) to form NiCo-MOF/AB (Fig. 7a, b). The assembled NiCo-MOF/AB║AC supercapattery device (Fig. 7c, d) revealed a specific capacitance of 115.05 F g^−1^ at a current density of 0.5 A g^−1^ and the maximum energy density of 33.84 Wh kg^−1^ as well as maximum power density of 15.1 kW kg^−1^.

Similarly, Yang et al. [189] designed a high-performance supercapattery device where graphene/Ni foam (GN) was utilized as substrate for the coating of carbon nanotube (CNT) wrapped Ni-MOF (Ni-MOF@CNT) to form the positive electrode Ni-MOF@CNT/GN. The Ni-MOF@CNT/GN-based solid-state supercapattery device was fabricated using activated carbon (AC) negative electrode. Tremendously, the fabricated device achieved high energy density of 135.84 Wh kg^−1^ with very good cyclic stability (93%) after 4000 cycles and higher performance was due to combined advantage of higher conductivity and high surface area of CNT along with porosity and unique structure of MOF. Polypyrrole nanotubes can help in uplifting the electronic conductivity and obstructing the nanosheets aggregation. Hence, Liu et al. [190] formed a polypyrrole nanotube (PNT) wrapped bimetallic MOF (NiCo-MOF) to form NiCo-MOF@PNT. Herein, the high porosity and ample redox active centers of MOFs availed a huge energy storage efficiency and PNT allowed good conductivity and impeded nanosheets aggregation. Therefore, maximum energy density of 41.2 Wh kg^−1^ with cyclic stability of 79.1% (10,000 cycles) was achieved in the fabricated electrode. Implementation of pure metal oxide may tend to increase the electrode resistance, which may avail low power density compared to the carbon-based EDLC materials. Therefore, the introduction of porous silver dendrites (Ag) along with highly conductive carbon nanotube (CNT) into bimetallic MOF (CuCo-MOF)-derived copper cobaltite (CuCo_2_O_4_) nanoflower to form CNT–CuCo_2_O_4_@Ag was a highly successful strategy by Vadiyar et al. [191]. The assembled supercapattery CNT–CuCo_2_O_4_@Ag║AC achieved an excellent energy density of 91 Wh kg^−1^, maximum power density of 18 kW kg^−1^ along with 98% cyclic stability for 20,000 cycles.

### MXene-Based Electrode Materials

In the search of novel electrode materials for electrochemical energy storage materials, researchers found a potential candidate named MXene, i.e., M_n+1_X_n_T_x_. The nomenclature can be described as follows: M is early transition metals like Ti, V, Sc or Cr, X is either carbon (C) or nitrogen (N), and T stands for many functional groups like O^−^, OH^−^, F^−^, etc. The value of n can be 1, 2 or 3 [192–194]. Therefore, MXenes are novel materials consisting of two-dimensional metal nitrides and carbides possessing excellent electrochemical efficiency owing to their special features like metal conductivity, surface hydrophilicity, etc. [194]. However, the maximum specific capacitance achieved by this material is nearly equal to 100 F g^−1^ which is too lower than the traditional carbon electrode materials. In order to eradicate the stacking and low specific capacitance, Zou et al. [195] hybridized 2D Ti_3_C_2_T_x_ with α-Fe_2_O_3_ and achieved specific capacitance 405.4 F g^−1^ at a current density of 1 A g^−1^ in negative electrode. Similarly, Malchik et al. [196] hybridized Mo_6_S_8_ with Ti_3_C_2_ to form Mo_6_S_8_/Ti_3_C_2_ electrode material whose working potential was − 1.1–0 V in 14 M LiCl. Li et al. [197] fabricated the modified MXene by removing the terminal group from pure MXene along with cation (K^+^) intercalation, which enhanced the specific capacitance value by 211%. The modified MXene (2D-Ti_3_C_2_) electrode delivered a specific capacitance of 517 F g^−1^ at a current density of 1 A g^−1^.

**Fig. 8:**
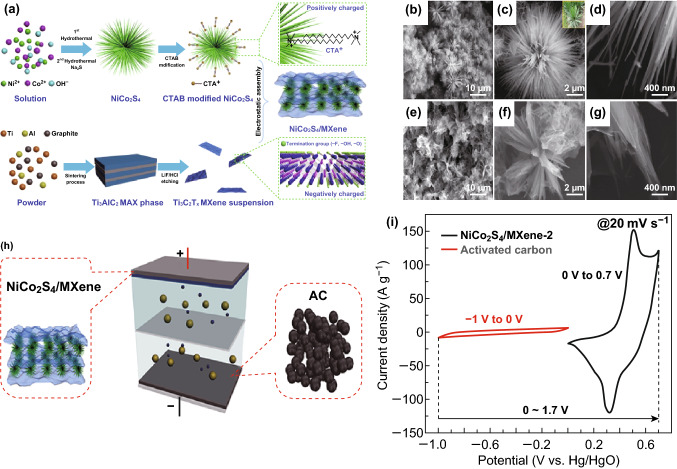
**a** Schematic illustration of detailed synthesis process of NiCo_2_S_4_/MXene composite. **b**–**d** SEM images of NiCo_2_S_4_ (sisal-like) and **e**–**g** MXene/NiCo_2_S_4_ composite at different magnifications. **h** Pictorial showing the model of supercapattery device NiCo_2_S_4_/MXene║AC, **i** individual CV curves of NiCo_2_S_4_/MXene and activated carbon at 20 mV s^−1^. Reprinted with permission from Ref. [199]. Copyright 2019 Elsevier

Further, abundant surface functional groups like fluorine (–F), hydroxyl (–OH) and oxygen (–O) may lead to the easy growth of various heterostructures on the surface of MXenes [198]. He et al. [198] synthesized nickel cobalt sulfide and MXene nanohybrid (Ni_1.5_Co_1.5_S_4_@Ti_3_C_2_). In this nanohybrid, metal sulfide (Ni_1.5_Co_1.5_S_4_) provided an good nanostructure, rich redox reactions along with high specific capacitance, whereas MXenes (Ti_3_C_2_) provided electrode wettability and electrical conductivity. A supercapattery was assembled using this Ni_1.5_Co_1.5_S_4_@Ti_3_C_2_ nanohybrid as positive electrode and activated carbon (AC) as negative electrode. This device demonstrated a maximum energy density of 49.84 Wh kg^−1^ and a maximum power density of 15.4 kW kg^−1^, respectively. Fu et al. [199] fabricated a MXene with bimetallic sulfide (NiCo_2_S_4_), i.e., NiCo_2_S_4_/MXene composite. This metal sulfide showed a sisal-like structure and over that MXene was grown (Fig. 8a–g). Addition of MXene with 3D architectured NiCo_2_S_4_ enhanced the cyclic stability of the material due to the presence of MXene, whereas NiCo_2_S_4_ provided rich redox active sites for electrochemical reaction. The NiCo_2_S_4_/MXene║AC supercapattery device (Fig. 8h, i) delivered a high energy density of 68.7 Wh kg^−1^ at a power density of 0.85 kW kg^−1^. Likewise, Li et al. [200] designed a supercapattery out of battery-type MXene-NiCo_2_S_4_ over nickel foam (NF) substrate to form the positive electrode (MXene-NiCo_2_S_4_@NF) and activated carbon (AC) as a negative electrode material. The device worked in the potential window of 1.6 V and obtained maximum power density of 3.38 kW kg^−1^. Wang et al. [201] prepared a nickel molybdate (NiMoO_4_) over Ti_3_C_2_T_x_ (MXene) nanosheets forming an interconnected porous network. The reduced graphene oxide hydrogel (rGH) was used as a negative electrode for the supercapattery, whereas NiMoO_4_/Ti_3_C_2_T_x_ nanosheets as a positive electrode. Without MXene, the device NiMoO_4_/rGH revealed 38.9% cyclic stability, whereas with MXene the device NiMoO_4_/Ti_3_C_2_T_x_║rGH retained 72.6% cyclic stability over 10,000 cycles.

Another group of material showing high electrochemical performance is layered double hydroxide (LDH), which possesses very high specific capacitance but poor electronic conductivity and feeble cyclic stability due to its tendency of structural aggregation [202, 203]. Additionally, MXenes hold high specific capacitance along with very good chemical and mechanical stability [204–206]. Therefore, taking the advantage of both kind of materials, Lu et al. [207] fabricated Ni_2_Co-LDH and Al-Ti_3_C_2_ MXene composite, i.e., Ni_2_Co-LDH@ Al-Ti_3_C_2_ MXene to fabricate a supercapattery device, wherein graphene hydrogel was implemented as a negative electrode material. The combined merits of LDH, MXene and graphene made the device to achieve a higher energy density of 68 Wh kg^−1^ with maximum power density of 20.3 kW kg^−1^. Niu et al. [208] synthesized another composite of CoAl-LDH and MXene, i.e., MXene/CoAl-LDH, which demonstrates high energy density of 30.9 Wh kg^−1^ with outstanding cyclic stability of 94.4% even after 30,000 cycles. LDHs are made up of positively charged metal hydroxides along with brucite-like layers with anions. Thus, their structure provides adequate surface redox reaction sites (pseudocapacitive), and in turn good electrochemical performance [209, 210]. However, their poor conductivity, low surface area and irreversible restacking nature [211, 212] are circumvented by the introduction of MXenes to them. In fact, the high metallic conductivity and excellent stability of MXene materials can be exploited to form different hybrid materials to get even better electrochemical performance in near future.

## Advancement in Supercapattery Research

The main objective of the supercapattery is to improve the energy density comparably higher than that of the existing supercapacitors with higher power density, longer life cycle than the benchmark Li-ion batteries. Therefore, cell design, materials selection, electrolyte and hybridization are key parameters to decide the final performance of supercapatteries. In this section, we consciously provided the performance of supercapatteries with various choices of materials. The performance of the supercapattery purely depends on the capacitance of the electrode, which is directly related to the active surface area, fast ion and electron transfer, low interfacial resistance and meso-/microporous structure. Moreover, extending the operating potential of supercapattery device is also another way of improving the performance by modifying the work function of both electrodes and types of electrolyte.

**Fig. 9:**
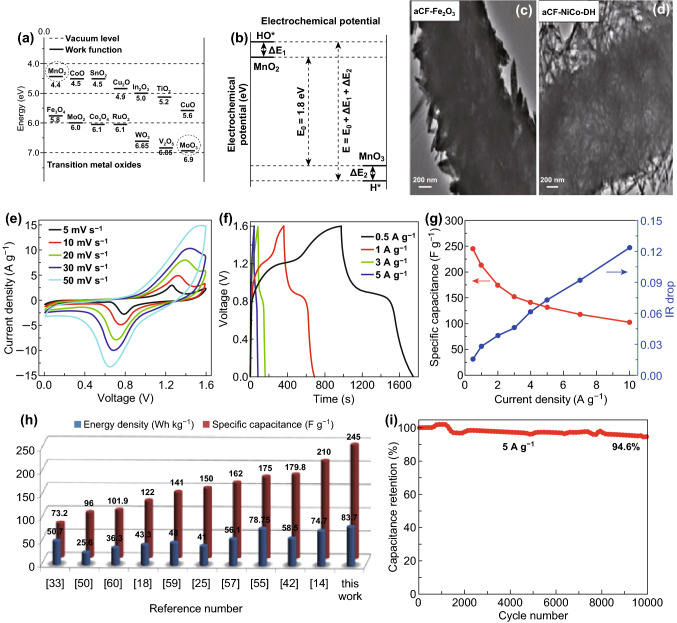
**a** Graphical representation of work function of various transition metal oxides. **b** The relationship between the electrochemical potential window and the shift of work function during charging process. Reproduced with permission from Ref. [81]. Copyright 2013 WILEY-VCH Verlag GmbH & Co. KGaA, Weinheim. TEM images of **c** aCF-Fe2O3 and **d** aCF-NiCo-DH active materials. **e** Cyclic voltammetry and **f** galvanostatic charge/discharge curves of the aCF-NiCo-DH║aCF-Fe2O3 asymmetric device in 2 M KOH aqueous electrolyte. **g** Specific capacitance and IR drop versus current density. **h** Comparison of energy density and specific capacitance with the previous reports. **i** Cyclic stability test of the aCF-NiCo-DH║aCF-Fe2O3 asymmetric device. Adapted with permission from Ref. [213]. Copyright 2016 Elsevier

Figure 9a shows a clear view about the work function of different metal oxide electrodes. This chart provides a clear picture about the selection of positive and negative electrodes to widen the full cell operating potential [81]. It can be obvious (Fig. 9a) that we could get the maximum potential window if we take the combination of MnO_2_ and MoO_3_. To verify this concept, Chang et al. [81] fabricated an asymmetric supercapacitor with the rGO–MnO_2_║rGO–MoO_3_ device in an aqueous electrolyte, and it delivered a high energy density of 42.6 Wh kg^−1^ and a power density of 276 W kg^−1^ with a specific capacitance of 307 F g^−1^. The pictorial representation of the relationship between potential window and the shift of the work function of both electrodes is given in Fig. 9b.

Various combinations of nanostructured carbon with redox-based materials (pseudocapacitive and battery type) are used for the construction of supercapattery device. Further, various materials and the performance of these supercapattery devices are detailed here as well as in Table 4. Li et al. [213] reported the synthesis of Fe_2_O_3_ nanorod and nickel cobalt double hydroxide (NiCo-DH) on activated conductive fiber as a negatrode and positrode, respectively (Fig. 9c–i). The reported device delivered an excellent energy density of 83.7 Wh kg^−1^ with a power density of 392.3 W kg^−1^ and the very high capacitance retention of 94.6% even after 10,000 cycles. Jana et al. [214] fabricated an asymmetric supercapacitor using conducting carbon cloth electrode as a negatrode and Ni–Co binary hydroxide/G as a positrode, which delivered an excellent energy density of 92 Wh kg^−1^ and power density of 7000 W kg^−1^ with 80% capacitance retention even after 10,000 cycles. Guo et al. [51] reported an asymmetric supercapacitor with Co_2_CuS_4_/graphene as a positrode and graphene nanosheets (NG) as a negatrode. The fabricated device showed an energy density of 53.3 Wh kg^−1^ and power density of 10,936 W kg^−1^ with an outstanding cyclic stability of 96.3% even after 5000 cycles. Balamurugan et al. [215] reported a cobalt-molybdenum sulfide nanosheets based asymmetric supercapacitor with Co–Mo–S NS as a positrode and N-doped graphene nanosheet as a negatrode. This device resulted energy and power densities of 89.6 Wh kg^−1^ and 20.07 kW kg^−1^, respectively, where capacitance retention is 86.8% after 50,000 cycles.

**Table 4:** Overview of electrochemical performance of various supercapatteries

Sl. no.	Electrode	Scan rate/current density	Electrolyte	Specific capacitance	Potential window	Energy density (max)	Power density (max)	Cyclic stability	Refs.
1.	Co_2_CuS_4_/graphene║graphene nanosheets (NG)	1 A g^−1^	6 M KOH	150 F g^−1^	1.6 V	53.3 Wh kg^−1^	10,936 W kg^−1^	96.3% after 5000 cycles	[51]
2.	rGO–MnO_2_║rGO–MoO_3_	200 mA g^−1^	1 M Na_2_SO_4_	307 F g^−1^	2.0 V	42.6 Wh kg^−1^	276 W kg^−1^	100% after 1000 cycles	[81]
3.	AC║urchin-like TiO_2_	0.5 A g^−1^	1 M LiPF_6_ in (EC) and (DMC)	61.2 F g^−1^	2.8 V	50.6 Wh kg^−1^	12.2 kW kg^−1^	87% after 5000 cycles	[89]
4.	AC║Li metal	NA	0.01 M LiClO_4_ + (BMPyrrFAP + γ-GBL)	NA	2.6 V	232 Wh kg^−1^	NA	NA	[93]
5.	CNT–CuCo_2_O_4_@Ag║AC	10 A g^−1^	PVA-KOH	159.7 F g^−1^	1.6 V	91 Wh kg^−1^	0.42 kW kg^−1^	98% after 20,000 cycles	[191]
6.	Ni_1.5_Co_1.5_S_4_@Ti_3_C_2_ (MXene)║AC	1 A g^−1^	2 M KOH	140 F g^−1^	1.6 V	49.84 Wh kg^−1^	15.47 kW kg^−1^	90% after 8000 cycles	[198]
7.	NiCo_2_S_4_/Ti_3_C_2_T_x_ MXene║AC	1 A g^−1^	3 M KOH	171.2 F g^−1^	1.7 V	68.7 Wh kg^−1^	8.5 kW kg^−1^	89.5% after 5000 cycles	[199]
8.	MXene-NiCo_2_S_4_@NF║AC	NA	PVA-KOH	NA	1.6 V	27.24 Wh kg^−1^	3.38 kW kg^−1^	NA	[200]
9.	NiMoO_4_/Ti_3_C_2_T_x_║rGH	0.5 A g^−1^	3 M KOH	240.1 F g^−1^	1.6 V	33.76 Wh kg^−1^	15.9 kW kg^−1^	72.6% after 10,000 cycles	[201]
10.	Ni_2_Co-LDH@ Al-Ti_3_C_2_ MXene║graphene hydrogel	2 A g^−1^	6 M KOH	140 F g^−1^	1.6 V	68 Wh kg^−1^	20.3 kW kg^−1^	90% after 10,000 cycles	[207]
11.	MXene/CoAl-LDH║MXene/graphene	NA	3 M KOH	NA	1.2 V	30.9 Wh kg^−1^	10 kW kg^−1^	94.4% after 30,000 cycles	[208]
12.	aCF-NiCo-DH║aCF–Fe2O3 nanorod	0.5 A g^−1^	2 M KOH	245 F g^−1^	1.6 V	83.7 Wh kg^−1^	392.3 W kg^−1^	94.6% after 10,000 cycles	[213]
13.	Ni–Co binary hydroxide/G║carbon cloth	2 A g^−1^	6 M KOH	340 F g^−1^	1.4 V	92 Wh kg^−1^	7000 W kg^−1^	80% after 10,000 cycles	[214]
14.	Co–Mo–S║N-doped graphene nanosheet (NGNS)	1 A g^−1^	3 M KOH	252 F g^−1^	1.6 V	89.6 Wh kg^−1^	20.07 kW kg^−1^	86.8% after 50,000 cycles	[215]
15.	Mesoporous Bi_2_O_3_ micro-sponge balls║graphite	2.25 A g^−1^	6 M KOH	24 F g^−1^	1.5 V	7 Wh kg^−1^	2040 W kg^−1^	80% after 5000 cycles	[216]
16.	AC║carbon coated CoFe_2_O_4_	1 mA cm^−1^	1 M KOH	9.5 mF cm^−1^	1.5 V	1.6 μWh cm^−1^	67.2 μW cm^−1^	75% after 11,000 cycles	[217]
17.	Polyaniline–SrTiO_3_║AC	0.2 A g^−1^	1 M KOH	NA	1.5 V	13.2 Wh kg^−1^	299 W kg^−1^	114% after 4000 cycles	[218]
18.	MWCNT–Co_3_O_4_–Ag║AC	0.2 A g^−1^	1 M KOH	NA	1.5 V	16.5 Wh kg^−1^	297.5 W kg^−1^	93.6% after 3000 cycles	[219]
19.	CMO-aH║AC	1 A g^−1^	6 M KOH	NA	1.6 V	18.89 Wh kg^−1^	1.06 kW kg^−1^	93% after 5000 cycles	[220]
20.	Co_3_O_4_–rGO/Ni foam║AC	5 A g^−1^	1 M KOH	60 F g^−1^	1.6 V	20 Wh kg^−1^	1200 W kg^−1^	94.5% after 10,000 cycles	[221]
21.	Co_2_(CO_3_)(OH)_2_ nanoflakes║AC	5 mV s^−1^	1 M KOH	91 F g^−1^	1.5 V	26.22 Wh kg^−1^	828 W kg^−1^	85% after 4000 cycles	[222]
22.	2D MnCo_2_O_4_ nanosheet║AC	5 mA cm^−1^	6 M KOH	366.4 F g^−1^	1.5 V	33.8 Wh kg^−1^	318.9 W kg^−1^	85% after 10,000 cycles	[223]
23.	NiCo–MnO_2_║C-FP	1 A g^−1^	1 M KOH	130.67 F g^−1^	1.5 V	48.83 Wh kg^−1^	896.88 W kg^−1^	96.78% after 10,000 cycles	[224]
24.	Spongy-like α-Ni(OH)_2_║AC	2 A g^−1^	6 M KOH	246 F g^−1^	1.4 V	49 Wh kg^−1^	696 W kg^−1^	87% after 2000 cycles	[225]
25.	rGO/TiO_2_║AC	1 A g^−1^	1 M KOH	347.5 F g^−1^	1.45 V	54.37 Wh kg^−1^	420.8 W kg^−1^	92% after 3000 cycles	[226]
26.	3D flowerlike sheets-assembled hydration nickel phosphate (N-90)║rGO	1 A g^−1^	3 M KOH	183.45 F g^−1^	1.5 V	25.48 Wh kg^−1^	750.02 W kg^−1^	84.23% after 1000 cycles	[227]
27.	Ni_3_(PO_4_)_2_–Ag_3_PO_4_║AC	0.5 A g^−1^	1 M KOH	91.25 F g^−1^	1.6 V	32.4 Wh kg^−1^	399.5 W kg^−1^	82% after 5000 cycles	[228]
28.	Cobalt phosphate║AC	0.9 A g^−1^	3 M KOH	107.7 F g^−1^	1.5 V	43.2 Wh kg^−1^	5.8 kW kg^−1^	68% after 10,000 cycles	[229]
29.	Co_3_(PO_4_)_2_.8H_2_O/NF║activated carbon/NF	5 mA cm^−2^	1 M NaOH	11.2 F cm^−2^	1.6 V	29.29 Wh kg^−1^	4687 W kg^−1^	77.9% after 1000 cycles	[230]
30.	CNF/NiCoP║CNF/NiCoP	1.5 A g^−1^	1 M KOH	269 F g^−1^	1.6 V	36 Wh kg^−1^	1.2 kW kg^−1^	100% after 25,000 cycles	[231]
31.	NiCo_2_S_2.2_Se_1.8_║AC	2 A g^−1^	6 M KOH	118.7 F g^−1^	1.6 V	39.6 Wh kg^−1^	1501 W kg^−1^	80.4% after 5000 cycles	[232]
32.	LiCoPO_4_║FeVO_4_	5 mV s^−1^	1 M KOH	73 F g^−1^	1.6 V	21 Wh kg^−1^	1326 W kg^−1^	139% after 1000 cycles	[234]
33.	Co_3_O_4_–rGO║AC	0.5 A g^−1^	PVA/KOH	109.1 F g^−1^	1.6 V	38.8 Wh kg^−1^	400 W kg^−1^	88% after 10,000 cycles	[235]
34.	NA-LDH║AC	NA	6 M KOH	NA	1.7 V	40.26 Wh kg^−1^	943 W kg^−1^	94.5% after 5000 cycles	[236]
35.	NiCo_2_S_4_/Co_9_S8 hollow spheres║AC	5 A g^−1^	3 M KOH	196 F g^−1^	1.6 V	96.5 Wh kg^−1^	0.8 kW kg^−1^	~99.9% after 10,000 cycles	[237]
36.	p-doped poly (aniline-co-m-anilicacid)║AC	10 mV s^−1^	NA	102 F g^−1^	NA	34.33 Wh kg^−1^	0.34 W kg^−1^	NA	[238]
37.	sGNS/cMWCNT/PANI║aGNS	1 A g^−1^	1 M H_2_SO_4_	107 F g^−1^	1.6 V	20.5 Wh kg^−1^	25 kW kg^−1^	91% after 5000 cycles	[240]
38.	GF-CNT@α-Fe_2_O_3_║GF-CoMoO_4_	14 A g^−1^	2 M KOH	115.5 F g^−1^	1.6 V	74.7 Wh kg^−1^	1400 W kg^−1^	95.4% after 50,000 cycles	[241]
39.	CoNi-layered double hydroxide/carbon nanotube║Fe_2_O_3_–graphene nanocomposite	0.5 A g^−1^	3 M KOH	252.4 F g^−1^	1.5 V	98 Wh kg^−1^	22,826 W kg^−1^	78% after 1000 cycles	[242]
40.	AC║Nb_2_O_5_@C	1 A g^−1^	EMIMBF4: LiTFSI = 1:1	46.8 F g^−1^	4.0 V	101 Wh kg^−1^	20.7 kW kg^−1^	83% after 8000 cycles	[243]
41.	AC║Li_4_Ti_5_O_12_	NA	1 M LiTFSI in PMPyrr-TFSI	NA	3.0 V	98 Wh kg^−1^	1.93 kW kg^−1^	91% after 1500 cycles	[244]
42.	Mesoporous carbon║LiMn_2_O_4_	10 mA g^−1^	(LiTFSI) + (EMITFSI)	50.7 F g^−1^	2.0 V	28 Wh kg^−1^	217.5 W kg^−1^	NA	[245]
43.	α-Co (OH)_2_║rGO aerogel	0.15 mA cm^−2^	[BMPyr^+^][DCA^−^]	18.2 F cm^−2^	2.0 V	NA	NA	93% after 1000 cycles	[246]
44.	TiO_2_–rGO║LiMn_2_O_4_	NA	1 M LiPF_6_ in (EC) and (DMC)	NA	2.15 V	258 Wh kg^−1^	NA	90% after 1000 cycles	[247]
45.	T-Nb_2_O_5_ MNSs/rGO║MC/rGO	0.05 A g^−1^	1.5 M LiPF_6_ in (EC) and (DMC)	45.9 F g^−1^	2.5 V	56 Wh kg^−1^	25.6 kW kg^−1^	82% after 4000 cycles	[248]
46.	AC║Nb_2_O_5_ NWs/rGO	0.2 A g^−1^	1.5 M LiPF_6_ in (EC) and (DMC)	44 F g^−1^	3.0 V	106 Wh kg^−1^	14 kW kg^−1^	NA	[249]
47.	Ni(OH)_2_/UGF (ultra-thin graphene foam)║a-MEGO	1 A g^−1^	6 M KOH	119 F g^−1^	1.8 V	6.9 Wh kg^−1^	44 kW kg^−1^	63.2% after 10,000 cycles	[250]
48.	MnCo_2_S_4_/NSG-48║NSG	1 A g^−1^	1 M KOH	204.6 F g^−1^	1.5 V	62.9 Wh kg^−1^	0.74 kW kg^−1^	NA	[251]
49.	NiCoP║N-doped carbon nanofiber	0.5 A g^−1^	1 M KOH	378 F g^−1^	1.6 V	56 Wh kg^−1^	533 W kg^−1^	NA	[252]
50.	Ni-S/1d-Ti_3_C_2_║1d-Ti_3_C_2_	NA	6 M KOH	NA	1.9 V	20 Wh kg^−1^	10 kW kg^−1^	71.4% after 10,000 cycles	[253]

Recently, Shinde et al. [216] reported a mesoporous Bi_2_O_3_║graphite supercapattery device with energy and power densities of 7 Wh kg^−1^ and 2040 W kg^−1^, respectively. Further, Ni, Co-based materials occupied a major part in the improvement of energy density with different compositions. Sankar et al. [217] prepared carbon coated cobalt ferrite spherical nanoparticles for supercapattery device, and it showed an energy and power densities of 1.6 μWh cm^−1^ and 67.2 μW cm^−1^ with capacitance retention of 75% over 11,000 cycles. Shahabuddin et al. [218] improved the energy density to 13.2 Wh kg^−1^ using layered material polyaniline–SrTiO_3_ nanocube composite. Iqbal et al. [219] constructed supercapattery device using MWCNT–Co_3_O_4_–Ag electrode, which improved the energy and power densities of 16.5 Wh kg^−1^ and 297.5 W kg^−1^, respectively, through the incorporation of highly conductive multi-walled carbon nanotubes (MWCNTs)-Ag network in the electrode material. Kim et al. [220] reported the mesoporous cobalt molybdate-based supercapattery device with energy and power densities of 18.89 Wh kg^−1^ and 1.06 kW kg^−1^, respectively.

Raj et al. [221] reported the Co_3_O_4_–rGO on Ni foam for supercapattery device, and this device obtained an energy density of 20 Wh kg^−1^ and power density of 1200 W kg^−1^ as well as 94.5% capacity retention after 10,000 cycles. Layered structure of a nanocomposite can avail reduction in volume expansion tending to obstruct the specific capacitance loss, whereas amorphous nature provides an efficient ion transport channel implying good charge storage due to disorder. Based on this concept, Sankar et al. [222] fabricated a supercapattery device with Co_2_(CO_3_)(OH)_2_ nanoflakes electrode, and it obtained a specific capacitance of 91 F g^−1^ with a energy density of 26.22 Wh kg^−1^ and a power density of 828 W kg^−1^. The 2D structured materials are good enough to deliver a high energy density due to its high surface area, conductivity and short diffusion length. Saravanakumar et al. [223] reported the porous 2D-MnCo_2_O_4_ nanosheet electrode with energy and power densities of 33.8 Wh kg^−1^ and 318.9 W kg^−1^, respectively.

**Fig. 10:**
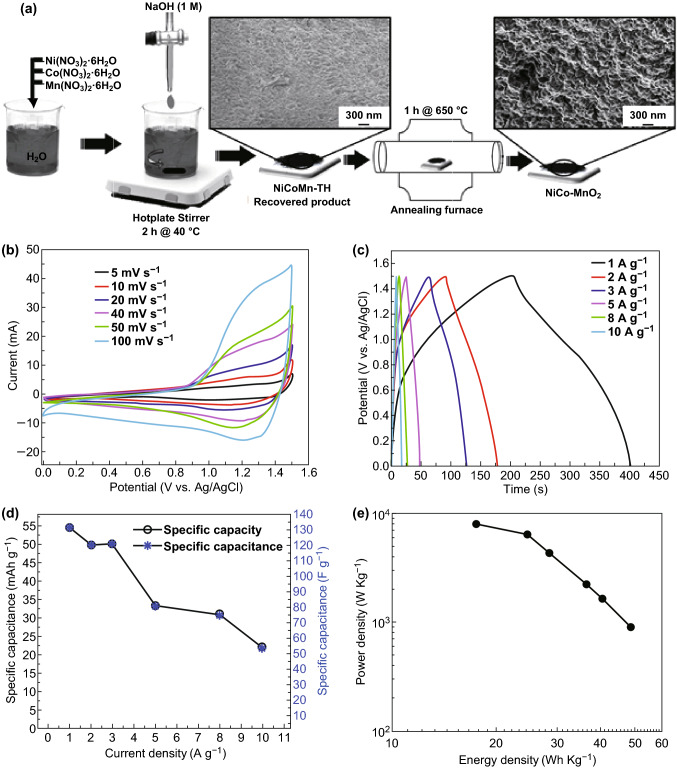
**a** Schematic representation of synthesis process of NiCo–MnO_2_ nanocomposite **b** CV and **c** galvanostatic charge/discharge curves of NiCo–MnO_2_║C-FP supercapattery device at a various scan rates and current densities. **d** Specific capacity/capacitance versus current density. **e** Ragone plots of the supercapattery device. Reprinted with permission from Ref. [224]. Copyright 2017 Elsevier

Oyedotun et al. [224] constructed a hybrid asymmetric supercapattery using NiCo–MnO_2_║C-FP electrodes (Fig. 10a–e), which yielded a specific capacitance of 130.67 F g^−1^ with an energy density of 48.83 Wh kg^−1^and a power density of 896.88 W kg^−1^. William et al. [225] reported a microwave-assisted synthesis of spongy-like α-Ni(OH)_2_ electrode for supercapattery application. The fabricated device resulted in a high energy density of 49 Wh kg^−1^ and a power density of 696 W kg^−1^. Similarly, other materials are also explored for the supercapattery application, which includes layered materials, transition metal sulfides and phosphites. Heng et al. [226] fabricated a supercapattery device based on reduced graphene oxide/titanium dioxide (rGO/TiO_2_) anode obtained a good energy density of 54.37 Wh kg^−1^ and a power density of 420.8 W kg^−1^ along with 92% capacity retention after 300 cycles. Peng et al. [227] reported a supercapattery with 3D flowerlike nickel phosphate (N-90) positive electrode showed a energy density of 25.48 Wh kg^−1^and a power density of 750.02 W kg^−1^. Omar et al. [228] devised a supercapattery device with Ni_3_(PO_4_)_2_–Ag_3_PO_4_ positive electrode, and this device delivered a specific energy density of 32.4 Wh kg^−1^ and a power density of 399.5 W kg^−1^ along with 82% capacity retention after 5000 cycles. Shao et al. [229] assembled a supercapattery device using cobalt phosphate. The fabricated device delivered a energy density of 43.2 Wh kg^−1^ at a power density of 5.8 kW kg^−1^. The same group [230] reported Co_3_(PO_4_)_2_.8H_2_O material on Ni foam with a gravimetric capacitance of 111.2 F g^−1^ and a specific energy and power densities of 29.29 Wh kg^−1^ and 4687 W kg^−1^, respectively. Another high-performing supercapattery device was fabricated by Surendran et al. [231] using nitrogen-doped carbon nanofiber and NiCoP material. The device showed a energy density of 56 Wh kg^−1^ and a power density of 533 W kg^−1^. A battery-type electrode material can avail fast ion diffusion path, poor charge transfer resistance as well as affluent electroactive sites resulting in high electrochemical efficiency. Such a battery-type electrode material, NiCo_2_S_2.2_Se_1.8_ nanotube array delivered energy density of 39.6 Wh kg^−1^ and power density of 1501.6 W kg^−1^ [232]. Lin et al. [233] synthesized a core–shell nanostructured NiCo_2_S_4_@Ni_3_S_2_ and applied as positive electrode for supercapattery device. The assembled device delivered a high energy density of 51.8 Wh kg^−1^ and a power density of 1039 W kg^−1^ along with 90.8% capacity retention after 5000 cycles.

Similarly, Nithya et al. [234] reported an LiCoPO_4_║FeVO_4_ asymmetric supercapacitor with an energy density of 21 Wh kg^−1^ and a power density of 1326 W kg^−1^. Lai et al. [235] designed a high-performance asymmetric supercapacitor (Co_3_O_4_–rGO║AC) which delivered a high energy density of 38.8 Wh kg^−1^ at a power density of 400 W kg^−1^with 88% capacitance retention even after 10,000 cycles. Zhang et al. [236] successfully developed an aqueous asymmetric supercapacitor with NA-LDH║AC and delivered high energy and power densities of 40.26 Wh kg^−1^ and 943 W kg^−1^, respectively, with 94.5% capacitance retention after 5000 cycles.

**Fig. 11:**
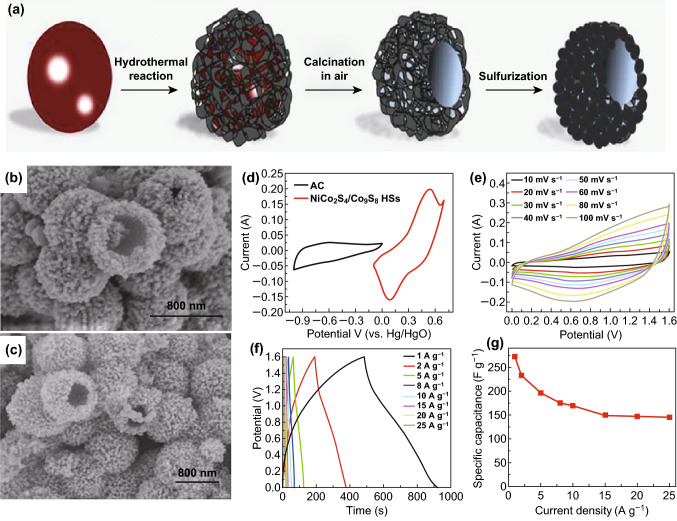
**a** Schematic illustration of detailed step by step synthesis of NiCo_2_S_4_/Co_9_S_8_ hollow spheres. **b**, **c** SEM image of NiCo_2_S_4_/Co_9_S_8_ from different angles showing hollow spheres. **d** Cyclic voltammetry curve of activated carbon and NiCo_2_S_4_/Co_9_S_8_ in one graph. **e** Cyclic voltammetry curve of supercapattery device (NiCo_2_S_4_/Co_9_S_8_║AC) at different scan rates. **f** Galvanostatic charge–discharge curve of supercapattery device at different current densities. **g** Rate capability of supercapattery device. Reproduced with permission from Ref. [237]. Copyright 2019 Elsevier

The morphology of as-prepared electrode material regulates the electrochemical performance of the device to a greater extent. Han et al. [237] fabricated a high-performance asymmetric supercapacitor utilizing NiCo_2_S_4_/Co_9_S_8_ hollow spheres as a positive and activated carbon (AC) as a negative electrode, which achieved a very high energy density of 96.5 Wh kg^−1^ at a power density of 0.8 kW kg^−1^ as shown in Fig. 11a–g. This unique hollow structure of NiCo_2_S_4_/Co_9_S_8_ material was having advantages such as lucid interior voids, low bulkiness, high surface area, appreciable pore volume and wettability, which leads to the excellent supercapattery performance.

Selvakumar and Pitchumani [238] reported a supercapattery with conducting polymer as a positrode based on p-doped poly (aniline-co-m-anilic acid) and activated carbon-coated material as a negatrode, which delivered the maximum specific capacitance of 102 F g^−1^. Laforgue et al. [239] constructed a hybrid supercapacitor device using poly (4-fluorophenyl-3-thiophene) or P-4-FPT as a positrode and an activated carbon as a negatrode. The fabricated device showed a maximum energy density of 48 Wh kg^−1^ with a power density of 9 kW kg^−1^. Shen et al. [240] fabricated an asymmetric high-performance supercapacitor based on nanoarchitectured sulfonated graphene nanosheet/carboxylated multi-walled carbon nanotube/polyaniline (sGNS/cMWCNT/PANI) as a positrode and activated graphene (aGNs) as a negatrode. This yields a high energy density of 20.5 Wh kg^−1^ at a power density of 25 kW kg^−1^. The rational design concept for metal oxide with carbon proved a quite effective way to improve the performance of the device. Guan et al. [241] reported an asymmetric supercapacitor using CoMoO_4_@graphite foam and Fe_2_O_3_@3D graphite foam–CNT forest hierarchical (GF-CNT@α-Fe_2_O_3_), which delivered a high specific capacitance of 210 F g^−1^ and a high energy density of 74.7 Wh kg^−1^ with a power density of 1400 W kg^−1^. Chen et al. [242] fabricated an asymmetric supercapacitor with porous Fe_2_O_3_–graphene nanocomposite and Co-, Ni-layered double hydroxide/carbon nanotube composite; it resulted in a high specific capacitance of 252.4 F g^−1^ with a tremendously high energy density of 98 Wh kg^−1^and a power density of 22,826 W kg^−1^.

In the above section of the manuscript, various aqueous electrolyte-based supercapatteries are described with their detailed performance and mechanisms. Researchers in the field of electrochemical energy storage have done many works on non-aqueous (organic and ionic) electrolyte-based supercapatteries. Some non-aqueous electrolyte-based supercapattery devices with their detailed performance are described below.

Zhang et al. [243] assembled a high-voltage supercapattery device opting niobium pentoxide (Nb_2_O_5_) nanoarray grown over graphene nanosheets (Nb_2_O_5_@C) as an anode and activated carbon as a cathode. This device covered a very high potential window of 4.0 V due to the use of ionic gel electrolyte (EMIMBF4: LiTFSI = 1:1) and the device obtained maximum energy density of 101 Wh kg^−1^ and power density of 20.7 kW kg^−1^ with cyclic stability of 83% after 8000 cycles. Fleischmann et al. [244] utilized lithium titanate (Li_4_Ti_5_O_12_) as an anode and activated carbon as a cathode to fabricate a non-aqueous supercapattery device. Use of this electrolyte provided a high operating potential window of 3.0 V with 91% cyclic stability after 1500 cycles. An excellent energy density of 98 Wh kg^−1^ and a maximum power density of 1.93 kW kg^−1^ were achieved by this device.

**Fig. 12:**
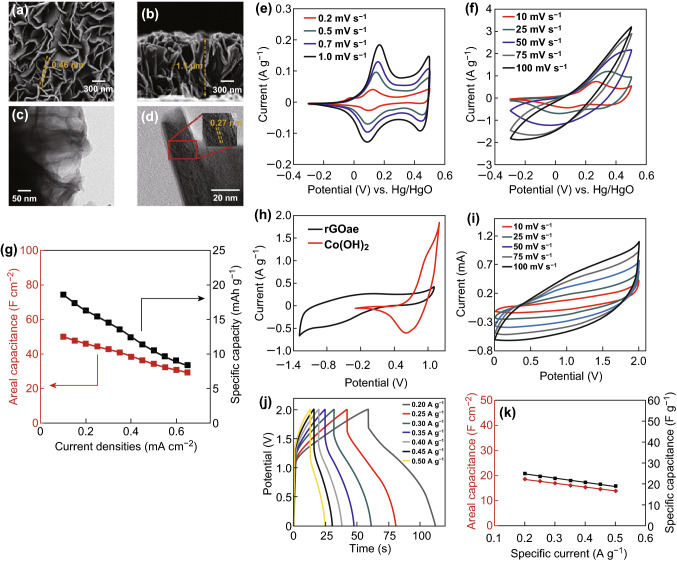
SEM image of **a** top view and **b** cross section view, **c** TEM and **d** HRTEM image of α-Co(OH)_2_ nanosheets. CV curves of α-Co(OH)_2_ nanosheets at **e** low scan rate and **f** high scan rate in 6 M KOH electrolyte. **g** Areal capacitance and gravimetric capacity of α-Co(OH)_2_ at different current densities. **h** Individual CV plots of α-Co(OH)_2_ and rGO aerogel. **i** CV, **j** GCD curve and **k** areal capacitance and gravimetric capacitance at various current densities of supercapattery device α-Co(OH)_2_║rGO_ae_. Adapted with permission from Ref. [246]. Copyright 2019 Elsevier

Junior et al. [245] fabricated a supercapattery using LiMn_2_O_4_ as an anode, mesoporous carbon as a cathode and 1 M lithium bis(trifluorosulfonyl)imide (LiTFSI)/1-ethyl-3-methylimidazolium bis(trifluoromethane) sulfonamide (EMITFSI) ionic electrolyte. Maximum energy density of this device was 20.8 Wh kg^−1^ with a maximum power density of 20.7 kW kg^−1^. Kongsawatvoragul et al. [246] utilized a thin film layered two-dimension α-Co (OH)_2_ material to design a high-performance supercapattery (Fig. 12a–k). Using 1-butyl-1-methyl-pyrrolidinium dicyanamide ([BMPyr^+^][DCA^−^]) ionic electrolyte, the device achieved 18.54 F cm^−2^ of areal capacitance at current density of 0.15 mA cm^−2^. The cyclic stability was 93% for 1000 charge discharge cycles. Kim et al. [247] designed a supercapattery device with TiO_2_–rGO║LiMn_2_O_4_ showing an excellent energy density of 258 Wh kg^−1^ with a cyclic stability of 90% for 1000 cycles. The operating potential window was 2.15 V in 1 M LiPF_6_ in ethylene carbonate (EC) and di-methyl carbonate (DMC) electrolyte.

Kim et al. [89] implemented activated carbon (AC) and urchin like TiO_2_ as positive and negative electrodes, respectively, and 1.5 M LiPF_6_ solution in 1:1 ethylene carbonate (EC): dimethyl carbonate (DMC) to make a high-efficient supercapattery. Maximum energy density obtained by this device was 50.6 Wh kg^−1^ with a maximum power density of 12.2 kW kg^−1^ and good cyclic stability of 87% even after 5000 cycles. Ma et al. [248] synthesized an orthorhombic phase Nb_2_O_5_ (T-Nb_2_O_5_) monodispersed mesoporous nanosphere (T-Nb_2_O_5_ MNSs) composited with reduced graphene oxide (rGO) (T-Nb_2_O_5_ MNSs/rGO) to use as a electrode material for supercapattery device. As positive electrode, this group introduced mesoporous carbon (MC)-coated rGO (MC/rGO) nanocomposite. In 1 M LiPF_6_ in ethylene carbonate (EC): dimethyl carbonate (DMC) electrolyte, this device covered a potential window of 2.5 V and achieved highest energy and power densities of 56 Wh kg^−1^ and 25.6 kW kg^−1^, respectively. Song et al. [249] prepared a flexible supercapattery device out of Nb_2_O_5_ nanowires composite with reduced graphene oxide (Nb_2_O_5_ NWs/rGO) anode and activated carbon (AC) as a cathode. Use of organic electrolyte 1.5 M LiPF_6_ solution in 1:1 ethylene carbonate (EC): dimethyl carbonate (DMC) provided this device not only with appreciable potential window of 3.0 V, but also tremendously high energy and power densities of 106 Wh kg^−1^ and 14 kW kg^−1^, respectively. Yu et al. [93] introduced 1-butyl-1-methylpyrrolidiniul tri(pentafluoroethyl)trifluorophosphate (BMPyrrFAP), gamma-butyrolactone (γ-GBL) and 0.01 M LiClO_4_ with excellent energy density of 232 Wh kg^−1^.

This section discussed the performance of some of the supercapatteries with various electrode materials. It gives clear idea of the development of new electrode materials (or) hybridization with novel materials for this application. Further, a rational design of the electrode is also another aspect to improve the performance of the device. The supercapattery is emerging as a new type of device. There is more research required in the view of materials selection for the positrode. For practical applications, a volumetric capacity is very important parameter compared to the gravimetric and areal capacities, so research should be focused on the development of high volumetric capacity electrodes. Most of the metal oxides and even ternary metal oxides are having insufficient electrical conductivity during the prolonged charge/discharge cycle; hence, it is necessary to hybridize them with highly conductive EDLC materials. But, in the case of EDLC materials, a low volumetric capacitance, which should be addressed by fabricating EDLC material with a higher surface area, mesoporous structure and replacing EDLC material with layered 2D pseudocapacitive materials like MXenes, MoS_2_, etc. In the view of redox active materials, it should be highly homogeneous in size distribution with fine nanoparticles, which allows high surface-active area, a short diffusion path length for ions/electrons and reduces the volume change during cycling. Further, more research is required in the view of selection of various electrolytes with wider operating potential window. Moreover, hybridizing various redox materials and nanostructured carbon materials (EDLC type) with distinct, innovative and effective morphologies and innovative device structure play a vital role in the performance of future supercapattery devices.

## Summary and Future Perspective

This review summarizes the necessity of supercapatteries from the existing energy storage devices like supercapacitors and batteries. The primary objective of this device is to improve the energy density with moderate power density through the hybridization of high-energy Faradaic electrodes with high power non-Faradaic electrodes. Various critical parameters involved in the energy storage mechanism have been discussed. Further, the electrochemical behavior of different electrode materials has been compared based on their charge storage mechanism. Moreover, this review article summarizes the bottleneck parameters of supercapatteries and provided suggestions to improve its performance. Perspectively, a carbon (EDLC type) and a redox-based material (pseudocapacitive and battery type) are the excellent choice of electrode materials for the supercapatteries to improve the energy and power densities. In this review, the electrochemical performance of the various types of carbon and its composite electrodes have been outlined. Mostly, the redox-based materials are good options for positive electrodes due to their high theoretical capacitance, which can improve the energy density. A various redox-based binary, ternary metal oxides, sulfides, phosphides and its composite based electrodes are highlighted here.

The future research trend should be focused on the following aspects to improve the performance of supercapatteries:

1. Synthesis of electroactive materials at a nanometer scale with high conductivity and porous structure could provide a higher surface area, shorter diffusion length for electrolyte ions. Hence, the development of new synthesis method is essential to prepare the electroactive materials at a nanometer scale with cost-effective and environment friendly manner.
2. Basically, carbon-based materials possess highly porosity with high surface area; hybridization of nanostructured carbon materials with redox-based electrode materials significantly improves the performance for supercapatteries. Hence, the research should focus on the development of new hybridized electrode materials without sacrificing the physical properties of carbon materials such as 3D structure, porosity and high electrical conductivity. Moreover, it is essential to select the redox materials with high electrochemical performance (capacity) for the hybridization process.
3. The electrolyte also plays a critical role to improve the supercapatteries performance. The development of electrolytes (aqueous and non-aqueous) with high ionic conductivity, energy, power densities with long cyclic stability as well as stable operation in elevated temperature range (− 40 to 85 °C) and relatively higher humidity is vital. Further, the selection and introduction of new type of redox-additive electrolyte is necessary to improve the potential window with safer operation zone, which could significantly increase the energy density of supercapatteries. The detailed research is required in the view of new aqueous electrolytes such as water-in-salt, hydrate-melt electrolytes for supercapatteries, which will ensure the high operating potential as well as safety.
4. The combination of positrode and negatrode for the supercapattery is very critical. So, it is important to select the positrode and negatrode with a high capacity, a wide operation potential, stable cycle life for long periods.
5. The current collector or electrode is also equally important in the contest. The development of lightweight, highly porous (micro/nanolevels), 3D architecture with high electrical conductivity, stability in various electrolytes and redox—active additives are vital parameters for the development of high-performance, lightweight supercapatteries.
6. In another view, the emerging new 2D materials such as metal dichalcogenides, MXenes, silicene, phosphene and so on are also good choice of materials for the hybridization with redox materials to enhance the performance of supercapattery devices through high electrical conductivity and high surface area to accommodate more redox materials.
7. Separator is another important parameter in supercapattery to improve the ionic conductivity. The development of new kinds of separator or surface modification is required for the safer and large operating potential window at elevated temperatures with different redox-active additives.
8. A considerable improvement is in the flexible, lightweight, high-energy supercapattery research, but still improvement is required to reach the consumer demand. So new electrode material development and device assembly should consider this point to achieve practically applicable high-energy supercapattery with an acceptable power.
9. The packing and compactness of the device are very critical for the implementation of supercapattery for real application. Therefore, researchers should focus more on the development of new packing and integration methods at low cost to accommodate supercapattery with other electronic devices without any issues.
10. Finally, more research should focus on the cost reduction of supercapatteries by developing low-cost fabrication methods, selection of high-performance, low-cost electrode materials and cost-effective packing techniques.

In conclusion, this review accomplishes that the electrode materials with high surface area, electrical conductivity, porous structure and the short ion/electron diffusion length are essential characteristics for the performance improvement of the supercapatteries. Moreover, it is important to develop new methods and materials to minimize the processing and manufacturing cost of the devices. The future research should be concentrated on the fabrication of supercapattery device by selecting nanostructured carbon materials and metal oxides with an excellent electrochemical performance. Researchers have achieved tremendously high values of energy and power densities for supercapatteries in comparison with supercapacitors and batteries using many different creative strategies. To meet out the modern society’s energy demand, further inventions are required to fabricate the cost-effective and environment friendly supercapatteries by means of facile synthesis and fabrication methods.
